# Artificial transmembrane signal transduction mediated by dynamic covalent chemistry†

**DOI:** 10.1039/d1sc04741h

**Published:** 2021-10-13

**Authors:** Carlo Bravin, Nol Duindam, Christopher A. Hunter

**Affiliations:** Yusuf Hamied Department of Chemistry, University of Cambridge Lensfield Road Cambridge CB2 1EW UK herchelsmith.orgchem@ch.cam.ac.uk

## Abstract

Reversible formation of covalent adducts between a thiol and a membrane-anchored Michael acceptor has been used to control the activation of a caged enzyme encapsulated inside vesicles. A peptide substrate and papain, caged as the mixed disulfide with methane thiol, were encapsulated inside vesicles, which contained Michael acceptors embedded in the lipid bilayer. In the absence of the Michael acceptor, addition of thiols to the external aqueous solution did not activate the enzyme to any significant extent. In the presence of the Michael acceptor, addition of benzyl thiol led to uncaging of the enzyme and hydrolysis of the peptide substrate to generate a fluorescence output signal. A charged thiol used as the input signal did not activate the enzyme. A Michael acceptor with a polar head group that cannot cross the lipid bilayer was just as effective at delivering benzyl thiol to the inner compartment of the vesicles as a non-polar Michael acceptor that can diffuse across the bilayer. The concentration dependence of the output signal suggests that the mechanism of signal transduction is based on increasing the local concentration of thiol present in the vesicles by the formation of Michael adducts. An interesting feature of this system is that enzyme activation is transient, which means that sequential addition of aliquots of thiol can be used to repeatedly generate an output signal.

## Abstract 2

Reversible formation of covalent adducts between a thiol and a membrane-anchored Michael acceptor has been used to control the activation of a caged enzyme encapsulated inside vesicles.

## Graphical abstract

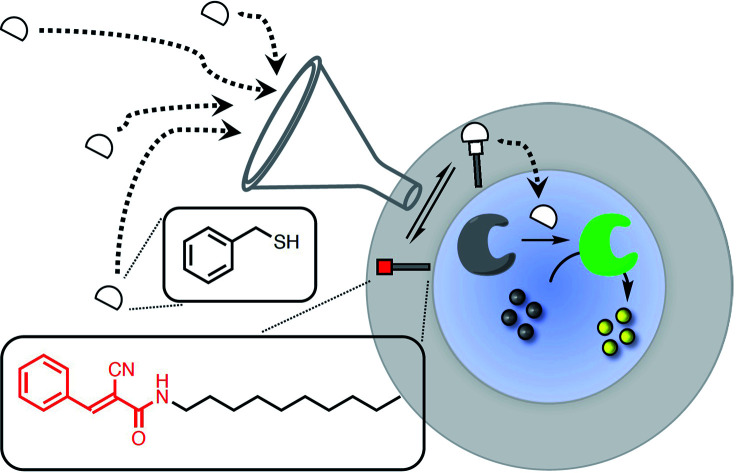

## Introduction

Many biological processes are based on compartmentalisation of chemical reactions. Vesicles provide the opportunity to develop synthetic systems that recapitulate some of the functional properties of cellular organisms, and in addition, there are potential biomedical applications, such as drug delivery.^1–5^ A key requirement for the development of proto-cellular entities is chemical communication between the internal and external compartments. Signalling processes across lipid bilayers can be activated through mechanisms involving either transduction of chemical signals^6–10^ or exchange of active components between the two sides of a membrane.^11–19^ Synthetic systems inspired by transmembrane protein receptors have been developed to control the generation of a compartmentalized signal in response to a specific molecular recognition event.^20–27^ In addition, dynamic covalent chemistry based on boronate esters,^28,29^ hemiaminals,^30^ and disulphides,^31–33^ has been used to mediate the transport of specific molecules across lipid bilayers. In this paper, selective transport across a bilayer is coupled with a compartmentalised catalytic process. We show that membrane-embedded Michael acceptors selectively enhance the transport of a thiol into synthetic vesicles. A caged enzyme, which is encapsulated on the inside of the vesicles, is activated by external addition of the thiol, but only when the Michael acceptor is present in the membrane. In addition, enzyme activation is transient in this system, so that the vesicles can be repeatedly triggered by sequential addition of multiple aliquots of thiol.

## Approach

**Fig. 1:**
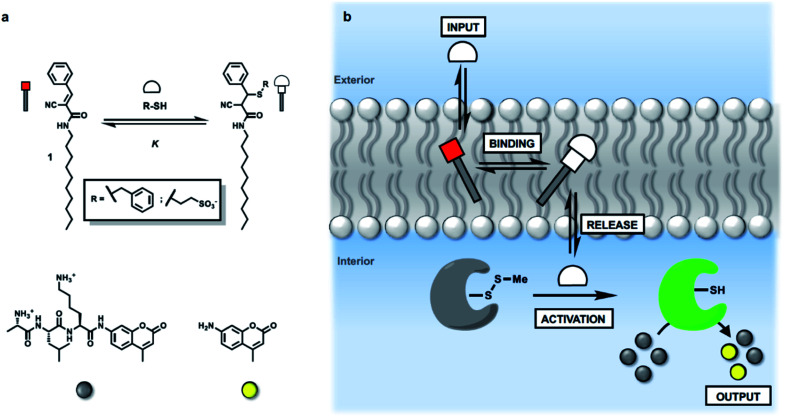
(a) Chemical structures of Michael acceptor **1**, the adduct formed by addition of a thiol, the peptide substrate for papain, and the fluorescent coumarin product obtained on peptide hydrolysis. (b) Activation of caged papain encapsulated in vesicles by addition of an external thiol (input signal). When a Michael acceptor is present in the membrane, there is a dynamic covalent interaction with the thiol. Binding and release of the thiol influences the ability of the thiol to penetrate the lipid bilayer and activate the caged papain in the internal compartment. Activation of papain leads to hydrolysis of an encapsulated peptide substrate and catalytic generation of an amplified output on the inside of the vesicle in the form of a fluorescence signal.

We have shown previously that membrane-embedded Michael acceptor **1** reacts reversibly with thiols added to the external aqueous solution (Fig. 1a).^34^ There is selectivity for different thiols, which is governed by partition of the thiol between the aqueous and lipid phases. For example, benzyl thiol formed the Michael adduct with an apparent binding constant (*K*) which is three orders of magnitude higher than the value measured for a charged thiol, 2-mercaptoethanesulfonate (see ESI S4.2†). Here we exploit this system to investigate whether the presence of **1** in a lipid bilayer membrane can be used to influence the internal response of an encapsulated enzyme to the external addition of thiols (Fig. 1b). Specifically, papain, which is caged by formation of the mixed disulfide of methane thiol, is encapsulated inside vesicles together with a peptide substrate (d-ala-leu-lys-7-amido-4-methycoumarin), which releases a fluorescent coumarin product when it is hydrolysed. If a thiol which is added to the external solution crosses the bilayer, disulfide exchange with the mixed disulfide that cages the papain active site cysteine will release the catalytically active thiol group on the protein. The active enzyme will then generate a fluorescent signal due to turnover of the substrate inside the vesicles.^35,36^

## Results and discussion

### Signal transduction triggered by reversible michael adduct formation

Synthetic vesicles, which contained the caged papain and the peptide substrate in the inner compartment, were first prepared. 1,2-Dioleoyl-*sn*-glycero-3-phosphocholine (DOPC) was mixed with a methanol solution of Michael acceptor **1** (10% mol loading) and the mixture was dried under nitrogen. The lipid film was rehydrated at pH 7.2 in HEPES buffer with the peptide substrate (250 μM) and the caged papain (10 μM). After extruding the suspension, vesicles with an average size of 400 nm were obtained with a bulk concentration of 1 mM lipid and 100 μM of **1** (for the full detailed procedure see ESI S3†). The presence of **1** and the peptide substrate in the vesicle suspension was confirmed by HPLC (see ESI S5†), and the presence of the enzyme was confirmed by the signalling experiments described below.

**Fig. 2:**
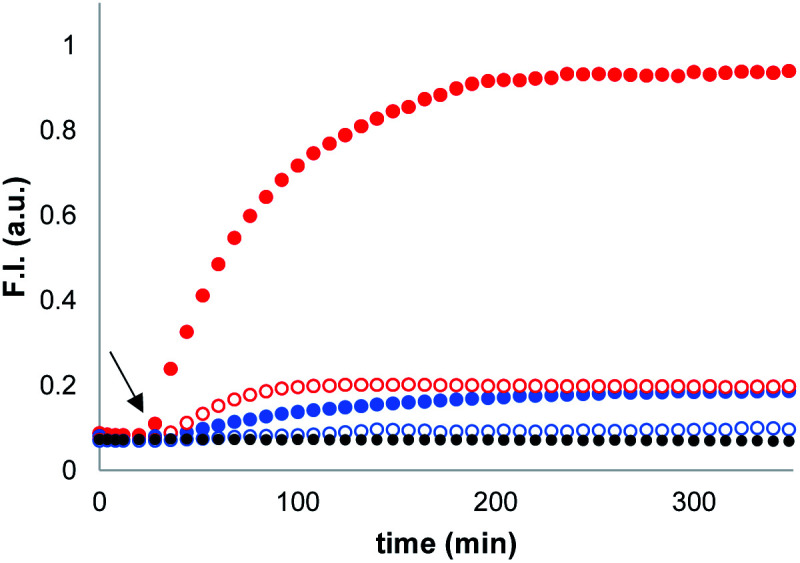
Time dependence of the normalised fluorescence emission intensity at 440 nm (*λ*_exc_ = 365 nm) from vesicle suspensions (DOPC, 1.0 mM) containing peptide substrate (250 μM) and caged papain (10 μM) in HEPES buffer (pH = 7.2). Thiols were added after 20 min (black arrow): addition of benzyl thiol (100 μM) to vesicles with 10% loading of **1** [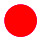] and without **1** [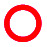]. Addition of 2-mercaptoethanesulfonate (100 μM) to vesicles with 10% loading of **1** [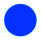] and without **1** [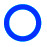]; no thiol added [●].

Signalling experiments were conducted by addition of benzyl thiol and 2-mercaptoethanesulfonate to two different batches of vesicles, which were prepared with or without Michael acceptor **1** present in the lipid mixture. Fluorescence due to the coumarin product obtained from enzyme-catalysed hydrolysis of the peptide substrate was monitored as function of time, and the results are shown in Fig. 2. Addition of the charged thiol (open blue circles) is almost indistinguishable from the background (black), which indicates that this thiol does not cross the lipid bilayer as expected. Addition of benzyl thiol, which is neutral and has a significantly higher membrane permeability, leads to a small increase in fluorescence over the first hour (open red circles), suggesting that this thiol is able to activate the caged papain to some extent. However, the presence of Michael acceptor **1** has a dramatic effect on the behaviour of the system. In the presence of **1**, there is a large increase in the intensity of the fluorescence signal obtained by adding benzyl thiol to the external aqueous solution (filled red circles). In contrast, **1** has very little effect on the signal obtained by adding the charged thiol (filled blue circles).

When these experiments were repeated in bulk solution in the absence of the lipid, the fluorescence response observed was similar for the two thiols (see ESI S6.2†), which shows that the difference observed in Fig. 2 is not related to an intrinsic difference in the reactivity of the two thiols with caged papain. The experiments were also repeated, but with the peptide substrate on the outside of the vesicles rather than on the inside. In this case, addition of thiol did not result in a fluorescent response. Subsequent addition of sodium cholate to disrupt the vesicles and bring the encapsulated enzyme into contact with the thiol and substrate triggered the hydrolysis reaction, and a rapid increase in fluorescence was observed (see ESI S6.1†). This experiment confirms that the bilayer effectively confines both the substrate and enzyme and that the vesicles are stable with respect to leakage in the presence of the thiol. We conclude that the behaviour shown in Fig. 2 must be related to the difference in the stability of membrane-embedded Michael adducts formed by the two thiols, which leads to selective transport across the bilayer.

### Transient enzyme activation

**Fig. 3:**
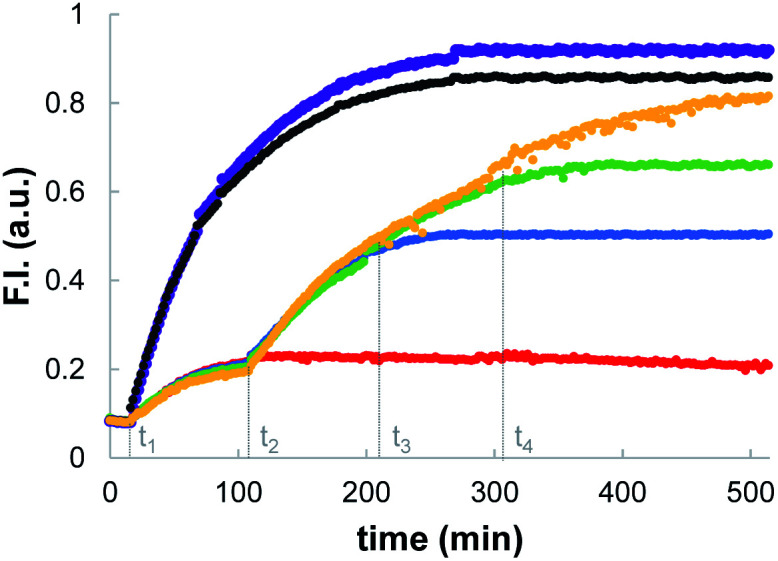
Time dependence of the normalised fluorescence emission intensity at 440 nm (*λ*_exc_ = 365 nm) after addition of aliquots of benzyl thiol to vesicles (DOPC, 1.0 mM) containing 100 μM 1 (10% loading), peptide substrate (250 μM) and caged papain (10 μM) in HEPES buffer (pH = 7.2). Benzyl thiol aliquots: 25 μM at *t*_1_ (red); 25 μM at *t*_1_ followed by 25 μM at *t*_2_ (blue); 25 μM at *t*_1_ followed by 25 μM at *t*_2_ and 25 μM at *t*_3_ (green); 25 μM at *t*_1_ followed by 25 μM at *t*_2_, 25 μM at *t*_3_ and 25 μM at *t*_4_ (orange); 100 μM at *t*_1_ (black); 200 μM at *t*_1_ (purple).

The data in Fig. 2 show that when the system is activated by addition of external thiol, the fluorescence initially increases but then plateaus after 1 to 2 hours. The plateau does not appear to be due to consumption of all of the substrate inside the vesicles. For example, the trace obtained for addition of benzyl thiol in the absence of Michael acceptor **1** in Fig. 2 shows that initially some of the papain was uncaged and started to hydrolyse the substrate. However, hydrolysis stopped at around 100 minutes, when only a fraction of substrate had been consumed compared with the result obtained in the presence of **1** (compare open and filled red circles). In a series of control experiments carried out in bulk solution without lipid, we found that when caged papain is activated by addition of benzyl thiol, the enzyme activity disappears after about 2 hours. Subsequent addition of a fresh aliquot of benzyl thiol can be used to restart catalytic hydrolysis of the substrate (see ESI S6.4†).^35,37,38^ This transient activation of papain was exploited in order to obtain a multi-step process in vesicles. Fig. 3 shows the results. Addition of large amounts of benzyl thiol (100–200 μM) lead to complete hydrolysis of all of the substrate encapsulated inside the vesicles (black and purple data). However, addition of smaller aliquots of benzyl thiol (25 μM) lead to the transient activation of the enzyme, and in each case, substrate hydrolysis stopped after a period of about 2 hours and could be reactivated by sequential addition of more thiol.

### Mechanism of signal transduction

**Fig. 4:**
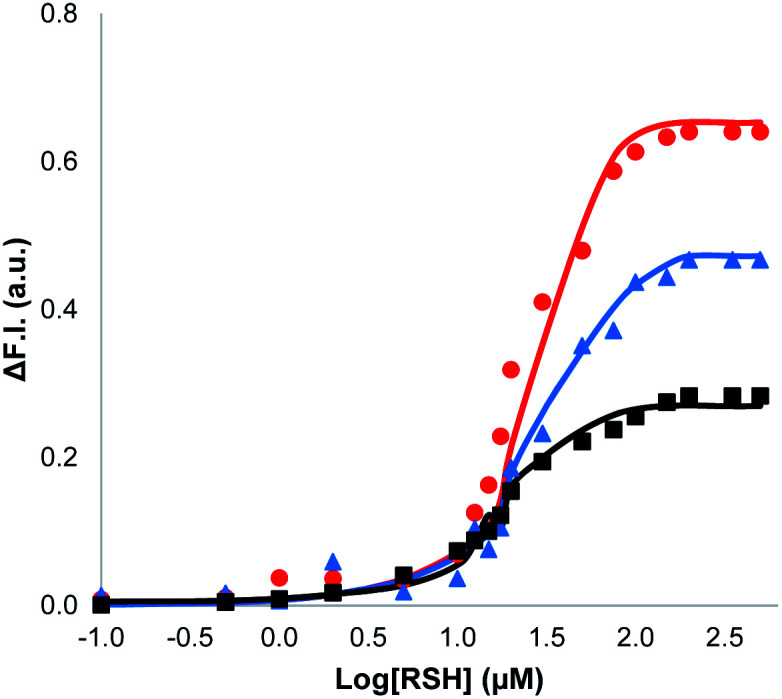
Change in normalised fluorescence emission intensity at 440 nm (*λ*_exc_ = 365 nm) (ΔF.I.) 360 min after addition of different concentrations of benzyl thiol ([RSH]) to vesicles (DOPC, 1.0 mM) containing peptide substrate (250 μM), caged papain (10 μM) and different loadings of **1** in HEPES buffer (pH = 7.2): 10% loading (red); 5% loading (blue); 1% loading (black). The lines are the best fit to an isotherm for equilibrium formation of a 1 : 1 adduct between the thiol and **1**.

The nature of the signalling process was investigated by using different loadings of **1** in the lipid bilayer and different concentrations of the benzyl thiol as the input signal (see ESI S6.3†). The output fluorescence signal was measured at 360 minutes, *i.e.* after the plateau had been reached in the response, and the results are summarised in Fig. 4. The intensity of the fluorescence signal increases with the loading of **1** in the membrane (black data 1%, blue data 5%, red data 10%). The fluorescence response also depends strongly on the thiol concentration: for concentrations less than 1 μM, no response was observed, and the response saturated at a concentration of about 100 μM. The data in Fig. 4 suggest that there is a saturation binding event that leads to formation of the active species, which determines the intensity of the signal obtained. The fluorescence data fit well to an isotherm for formation of a 1 : 1 adduct between the thiol and **1** (calculated lines shown in Fig. 4), and the resulting equilibrium constant of (1.0 ± 0.3 × 10^5^ M^−1^) is similar to the value measured previously by direct titration (2.0 ± 0.2 × 10^5^ M^−1^).^34^ The data in Fig. 4 show that the intensity of the fluorescence response obtained from this system is directly proportional to the concentration of Michael adduct formed in the lipid bilayer membrane. This result also explains why the response obtained with the charged thiol is so low: the equilibrium constant for formation of the Michael adduct with 2-mercaptoethanesulfonate is three orders of magnitude lower than the adduct formed with benzyl thiol, so the charged Michael adduct is not populated.

**Fig. 5:**
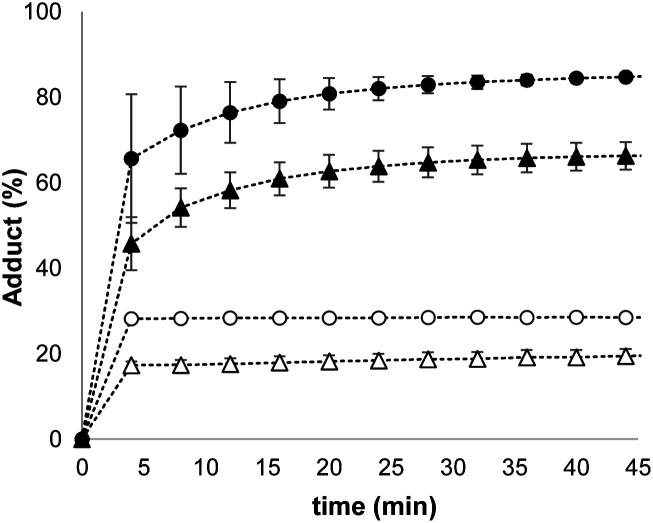
Time course for conversion of **2** (50 μM) embedded in vesicles (DOPC, 0.5 mM in HEPES buffer at pH 7.2) into the corresponding Michael adduct on addition of different thiols: 100 μM benzyl thiol [▲], 200 μM benzyl thiol [•], 5.0 mM 2-mercaptoethanesulfonate [Δ],10 mM 2-mercaptoethanesulfonate [o].

The results in Fig. 4 suggest that one possible mechanism of action for **1** is transport of the thiol across the lipid membrane, because the rate of transport would be proportional to the concentration of the Michael adduct. Thus, Michael acceptor **1** would react with the thiol on the outer surface of the vesicles and then cross the bilayer to release the thiol into the interior compartment. In order to test this hypothesis, we synthesised a second Michael acceptor, **2**, which has a polar head group and so cannot cross the lipid bilayer.^39^ Compound **2** was prepared in two steps by a Koenigs–Knorr glycosidation,^40,41^ followed by a Knoevenagel condensation. The interaction of thiols with **2** embedded in vesicles was first investigated using UV-Vis spectroscopy. Both benzyl thiol and 2-mercaptoethanesulfonate form the corresponding Michael adduct with **2**, and Fig. 5 shows that the adduct formed with benzyl thiol is significantly more stable than the adduct formed with the charged thiol. Titration experiments were used to determine the equilibrium constants *K* for formation of the Michael adducts of **2** in vesicles: 3.0 ± 0.3 × 10^4^ M^−1^ for benzyl thiol, and 50 ± 3 M^−1^ for the charged thiol (see ESI S4.2†). These values are about an order of magnitude lower than the values measured for formation of Michael adducts with **1**, but the behaviour is similar. The relative stability of the Michael adducts are determined by partitioning of the thiols between the aqueous and lipid phases.

**Fig. 6:**
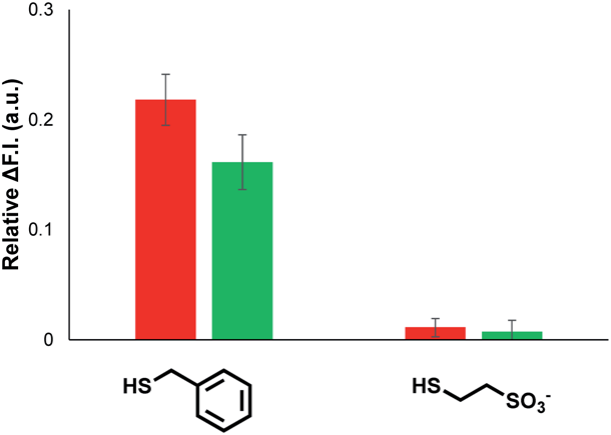
Change in normalised fluorescence emission intensity at 440 nm (*λ*_exc_ = 365 nm) (ΔF.I.) 360 min after addition of two different thiols (100 μM) to vesicles (DOPC, 1.0 mM) containing peptide substrate (250 μM), caged papain (10 μM) and either **1** (red) or **2** (green) at 1% loading (10 μM) in HEPES buffer (pH = 7.2).

**Fig. 7:**
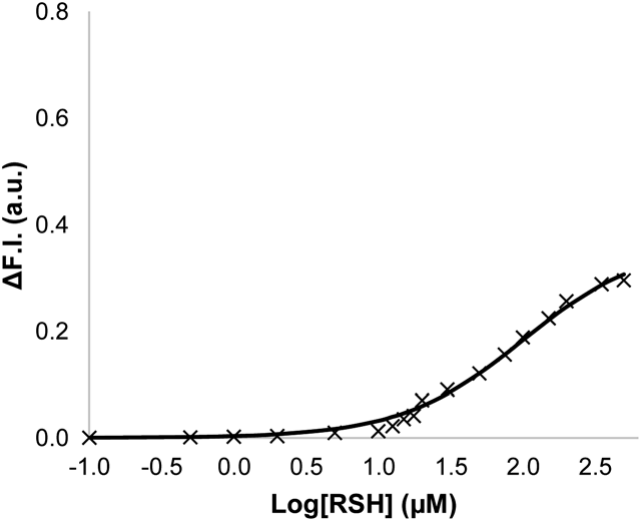
Change in normalised fluorescence emission intensity at 440 nm (*λ*_exc_ = 365 nm) (ΔF.I.) 360 min after addition of different concentrations of benzyl thiol ([RSH]) to vesicles (DOPC, 1.0 mM) containing peptide substrate (250 μM), caged papain (10 μM) and **2** (10 μM, 1% loading) in HEPES buffer (pH = 7.2). The line is the best fit to an isotherm for equilibrium formation of a 1 : 1 adduct between the thiol and **2**.

Signalling experiments were then conducted by external addition of thiols to vesicle suspensions containing Michael acceptor **2** embedded in the lipid membrane and caged papain and the peptide substrate encapsulated in the internal compartment. Fig. 6 compares the activity of the two different Michael acceptors in the signalling experiment under identical conditions. The behaviour of Michael acceptor **2** is practically identical to **1**. Addition of benzyl thiol to vesicles containing **2** leads to activation of caged papain and hydrolysis of the peptide substrate in the interior compartment of the vesicles. Addition of the charged thiol, 2-mercaptoethanesulfonate, to vesicles containing **2** does not activate the enzyme. We conclude that the signal transduction process does not involve the Michael adduct crossing the lipid bilayer to deliver the thiol to the inner compartment. Fig. 7 shows the effect of changing the concentration of benzyl thiol used as the input signal on the fluorescence output signal in the transduction process mediated by **2**. No output signal was observed below a thiol concentration of 10 μM, and the response began to saturate as the concentration of benzyl thiol approached 1 mM. These data are consistent with a saturation binding event that determines the intensity of the output signal, and fitting to an isotherm for formation of a 1 : 1 adduct between benzyl thiol and **2** (calculated line in Fig. 7) gave equilibrium constant of 1.0 ± 0.3 × 10^4^ M^−1^ which is in agreement with the value obtained by UV-Vis titration.

These results show that the formation of Michael adducts between the transducers and benzyl thiol is required for activity, but that the process of delivering thiol from the outer aqueous solution to the inner compartment does not require the Michael adduct to cross the bilayer. The role of the Michael acceptors appears to be simply to provide a mechanism for increasing the local concentration of thiols. Assuming that the binding and release of thiols from the Michael acceptors is fast and that benzyl thiol can cross the bilayer independently, then accumulation of benzyl thiol in the lipid bilayer appears to be responsible for uncaging the encapsulated enzyme.

## Conclusions

A new mechanism for transmembrane signal transduction is described. Caged papain was encapsulated inside vesicles along with a peptide substrate. Addition of thiols to the external aqueous solution did not activate the enzyme, even when a membrane permeable input signal, benzyl thiol, was used. However, when a Michael acceptor was embedded in the lipid bilayer membrane, external addition of benzyl thiol led to uncaging of the enzyme and hydrolysis of the peptide substrate, resulting in a fluorescent output signal. The system is selectively activated by benzyl thiol, and addition of a charged thiol, 2-mercaptoethanesulfonate, did not result in an output signal. The signalling activity is directly related to the concentration of the Michael adduct formed between the thiol and the membrane-anchored Michael acceptor. However, the location of the Michael acceptor in the membrane did not affect transmission of the signal across the bilayer. Thus a Michael acceptor, which was constrained to sit at the aqueous interface by a polar head group, was just as effective at delivering benzyl thiol to the internal compartment of the vesicles as a non-polar Michael acceptor, which can freely diffuse across the bilayer. The results presented here suggest that reversible formation of Michael adducts between the thiol input signal and membrane-anchored Michael acceptors leads to an increase in the local concentration of thiol in the vesicles, and this concentrator mechanism is responsible for enzyme activation. Activation of the enzyme in this system is transient, which means that sequential addition of aliquots of benzyl thiol can be used to repeatedly activate the enzyme and generate new fluorescence output signals.

## Data availability

All supporting data is provided in the ESI.†

## Author contributions

CAH and CB devised the experiments, CB carried out the experiments on transducer **1**, ND carried out the experiments on transducer **2**, and all authors contributed to the final manuscript.

## Conflicts of interest

There are no conflicts to declare.

## Supplementary Material

SC-012-D1SC04741H-s001
