# Supplementary material for: Artificial transmembrane signal transduction mediated by dynamic covalent chemistry
Source: Chem Sci. 2021 Oct 13;12(42):14059–64. doi: 10.1039/d1sc04741h (PMC8565364; doi:10.1039/d1sc04741h)
Supplement: SC-012-D1SC04741H-s001 [file SC-012-D1SC04741H-s001.pdf]

Electronic Supplementary Material (ESI) for Chemical Science.
This journal is © The Royal Society of Chemistry 2021

Artificial transmembrane signal transduction mediated by dynamic covalent chemistry

Carlo Bravin, Nol Duindam and Christopher A. Hunter *

Yusuf Hamied Department of Chemistry, University of Cambridge, Lensfield Road, Cambridge CB2 1EW (UK)

Supplementary Information

Contents

1 General Methods 2
2 Synthesis and Characterization 3
2.1 Intermediate 1 3
2.2 Michael acceptor 1 3
2.3 Intermediate S2 3
2.4 Michael acceptor 2 5
3 General procedure for vesicles preparation 8
3.1 Loaded vesicles with 2 for UV-Vis titration and binding constant determination 8
3.2 Papain (and) peptide encapsulation for signalling experiments 8
4 Kinetic Experiments and Binding Constant Determination for acceptor 2 9
4.1 UV-Vis spectra of thiol addition in Vesicles and Methanol 9
4.2 Kinetic profile of the 2 adduct formation and binding constant determination 10
4.2.1 Michael acceptor 2 kinetic traces in vesicles 12
4.2.2 Michael acceptor 2 kinetic traces in Methanol 13
5 Loaded Vesicles Preparation 14
5.1 Purification of enzyme and peptide encapsulated vesicles 14
6 Signalling experiment controls 15
6.1 Assessment of vesicles leakage upon thiols addition 15
6.2 Reactivity between different thiols and enzyme 16
6.3 System response with thiols 17
6.4 Signalling experiment at different loading of Michael acceptors and binding constant evaluation of thiol 3a 19
6.5 Papain deactivation 22
6.6 Multi step papain activation 24
References 25

1 General Methods

^{1}H NMR and ^{13}C NMR spectra were recorded on a 700 and 400 MHz Bruker spectrometer. Chemical shifts are reported as δ values in ppm. All the ^{1}H NMR spectra were referenced to residual isotopic impurity of $CDCl_3$ (7.26 ppm) and DMSO-d_6 (2.50 ppm). ^{13}C NMR spectra (100 MHz) were referenced to the $CDCl_3$ peak (77.0 ppm) and DMSO-d_6 (39.5 ppm). The following abbreviations are used in reporting the multiplicity for NMR resonances: s=single, d=doublet, t= triplet, and m=multiplet. The NMR data were processed using MestReNova 10.0.2.
High resolution electrospray ionization mass spectrometry (HRMS-ESI) was performed on Waters LCT Premier TOF Spectrometer or by the Mass Spectrometry Service at the Department of Chemistry. The LCMS analysis of samples was performed using Waters Acquity H-class UPLC coupled with a single quadrupole Waters SQD2. ACQUITY UPLC BEH C8 Column, 130Å, 1.7 µm, 2.1 mm X 50 mm was used as the UPLC column. The conditions of the UPLC method are as follows: Solvent A: Water +0.1% Formic acid; Solvent B: Acetonitrile +0.1% Formic acid; Gradient of 0-4 minutes 5% - 100%B + 1 minute 100% B with re-equilibration time of 2 minutes. Flow rate: 0.4 ml/min; Column temperature of 40 °C.
Melting point measurements were performed on Mettler Toledo MP90. Infrared (IR) spectra were recorded on Bruker Alpha. FTIR Spectrometer with single reflection diamond Platinum ATR. UV-Vis spectra were recorded using a Cary 60 (Agilent Technologies) in Hellma Analytics Suprasil quartz cuvettes.
Fluorescence measurement were performed using a micro-plate reader BMG CLARIOstar-430-0157; Software version 5.01 R2. The volume in each well was 150µL and the gain for the fluorescence emission of the coumarine dye fluorescence emission intensity at 440 nm (λ_{exc} = 365 nm) was 1200 for each measurement. All the measurement were repeated from three different preparations in duplicate. Fluorescence intensity was recorded every 2 minutes.
pH measurements were conducted using a Mettler-Toledo "Seven Compact" pH meter equipped with an "In-lab Micro" electrode. GPC purification of vesicles was carried out using Sephadex G-100 with a manual column.
Vesicles were prepared using Avestin "LiposoFast" extruder apparatus, equipped with polycarbonate membranes with 200 nm pores. Lipids were purchased from Sigma Aldrich and used without further purification. All the reagents and solvents were purchased from Sigma Aldrich and Acros and used without further purification. Deactivated Papain-SSMe was purchased from Thermo Fisher Scientific (Life Technologies) as part of the Thiol and Sulfide Quantitation kit T6060.

2 Synthesis and Characterization

2.1 Intermediate 1

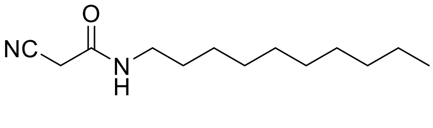

S1

S1 was synthesized according to the procedure reported in C. Bravin and C.A. Hunter, *Chem. Sci.*, **2020**, *11*, 9122-9125.

2.2 Michael acceptor 1

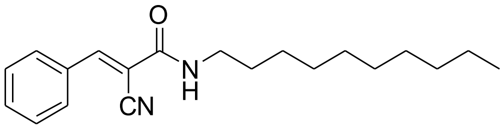

1

1 was synthesized according to the procedure reported in C. Bravin and C.A. Hunter, *Chem. Sci.*, **2020**, *11*, 9122-9125.

2.3 Intermediate S2

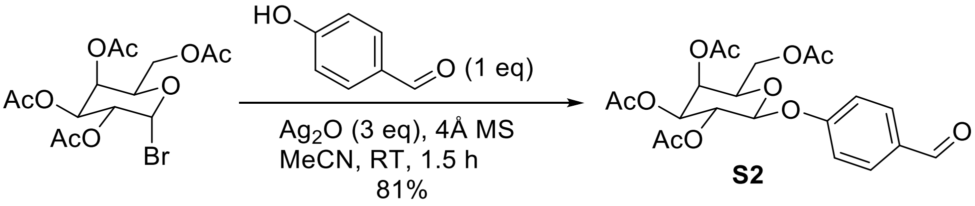

In a flame-dried flask acetobromo-α-D-galactose (500 mg, 1.22 mmol, 1 eq) and 4-hydroxybenzaldehyde (148 mg, 1.22 mmol, 1 eq) were dissolved in anhydrous MeCN (7.5 mL) under inert atmosphere. Subsequently activated <5 micron powdered 4Å molecular sieves (1.3 g) and Ag_2O (845 mg, 3.65 mmol, 3 eq) were added to the murky white solution. The resulting black suspension was stirred at RT for 1.5 hour, after which TLC indicated complete conversion of the starting material (eluent 40% EtOAc in PET, $R_{f,2}$=0.25, $R_{f,4\text{-hydroxybenzaldehyde}}$=0.55). The reaction mixture was filtered over a celite pad. The celite pad was subsequently flushed with EtOAc. The filtrate was concentrated *in vacuo* to yield 0.58 g of the crude product. The crude was then further purified by column chromatography on silica gel (0 – 40% EtOAc in PET), affording sugaraldehyde **S2** as a colourless oil (447 mg, 81% yield).

m.p.: oil at r.t.

^{1}H-NMR (400 MHz, $CDCl_3$): δ 9.92 (s, 1H), 7.85 (d, *J* = 8.7 Hz, 2H), 7.11 (d, *J* = 8.7 Hz, 2H), 5.51 (dd, *J* = 10.5, 7.9 Hz, 1H), 5.47 (d, *J* = 2.7 Hz, 1H), 5.17 (d, *J* = 7.9 Hz, 1H), 5.13 (dd, *J* = 10.5, 3.4 Hz, 1H), 4.29 – 4.04 (m, 3H), 2.18 (s, 3H), 2.06 (s, 6H), 2.01 (s, 3H).

^{13}C-NMR (100 MHz, $CDCl_3$): δ 190.79, 170.44, 170.28, 170.19, 169.42, 161.44, 132.01, 131.95, 116.92, 98.80, 71.52, 70.83, 68.57, 66.91, 61.50, 20.83, 20.79, 20.77, 20.69.

HRMS (ESI-TOF) (*m/z*): $[M+Na]^+$ calcd. for $[C_{21}H_{24}O_{11}+Na]^+$, 475.1216; found 475.1210

FT IR (KBr) ν (cm^{-1}): 1749, 1695, 1602, 1584, 1508, 1370, 1227, 1161, 1126, 1076

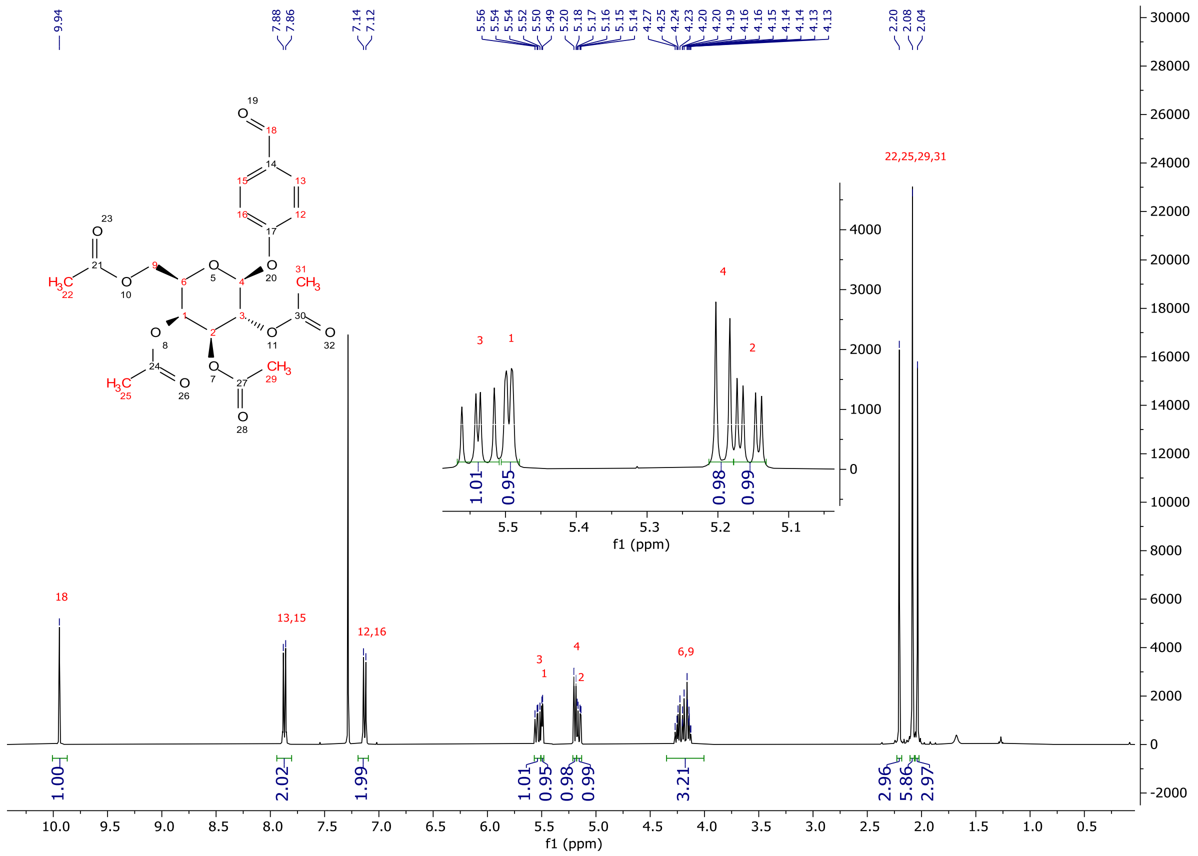

Figure S1 ^{1}H NMR (400 MHz, 301 K, $CDCl_3$) of compound **S2**

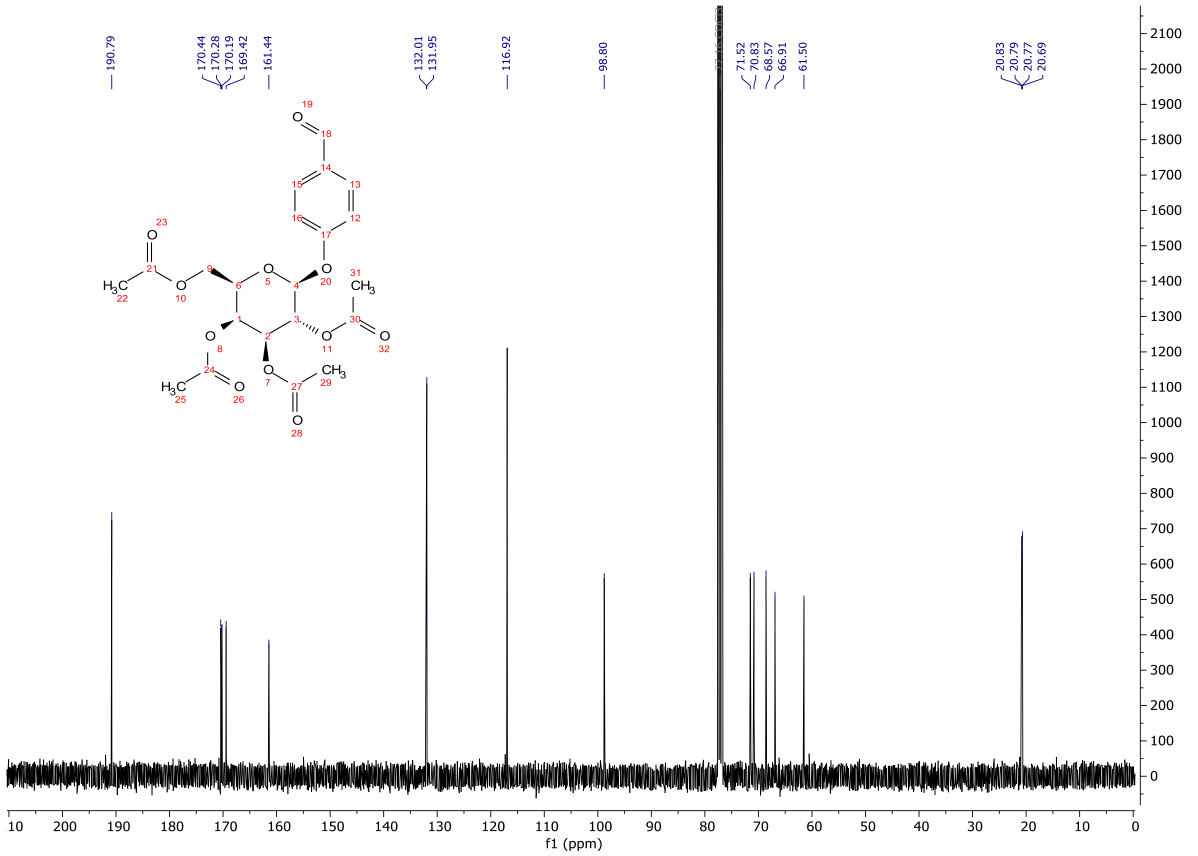

Figure S2 ^{13}C-NMR (100 MHz, 301 K, $CDCl_3$) of compound **S2**

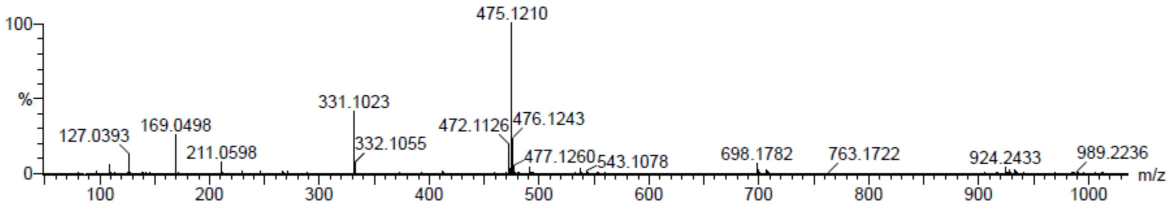

Figure S3 HRMS spectrum (ESI-TOF) (*m/z*) of **S1**

2.4 Michael acceptor 2

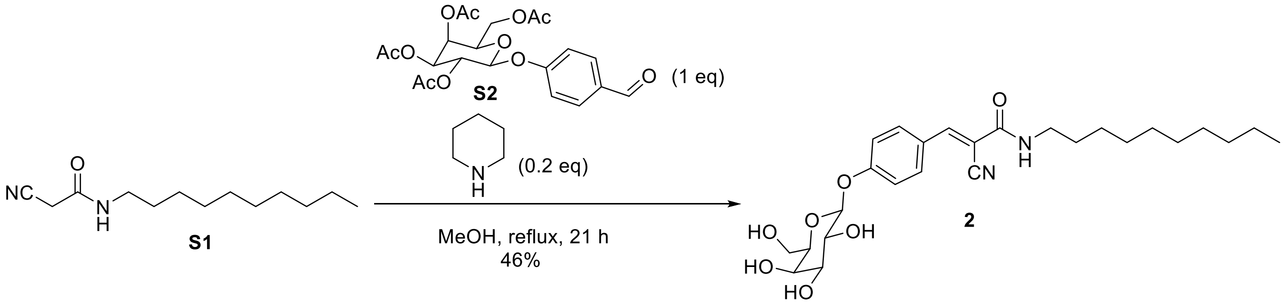

In a round bottom flask cyanoacetamide **S1** (50.5 mg, 225 µmol, 1 eq) and sugaraldehyde **S2** (102 mg, 225 µmol, 1 eq) were dissolved in 5 mL MeOH under inert atmosphere. To the solution 5 µL piperidine (4.3 mg, 51 µmol, 0.2 eq) was added. The reaction mixture was refluxed for 21 hours, after which TLC indicated complete conversion of the starting material (eluent 10% MeOH in DCM, $R_{f,2}$=0.70, $R_{f,3}$=0.30). The solution turned pale yellow over time and white precipitate was formed. The mixture was concentrated under reduced pressure and the resulting yellow powder was purified by column chromatography on silica gel (0 – 10% MeOH in DCM) affording **2** as a white powder (86 mg, 77% yield). The resulting powder was subsequently washed with acetone (3 x 4 mL) to afford **2** (51.3 mg, 46% yield).

m.p.: 189-195.8 ºC

^{1}H-NMR (700 MHz, DMSO-d$_6$): δ 8.33 (t, *J* = 5.7 Hz, 1H), 8.09 (s, 1H), 7.95 (d, *J* = 8.9 Hz, 2H), 7.20 (d, *J* = 8.9 Hz, 2H), 5.23 (d, *J* = 5.2 Hz, 1H), 4.98 (d, *J* = 7.7 Hz, 1H), 4.89 (d, *J* = 5.7 ,Hz, 1H), 4.66 (t, *J* = 5.5 Hz, 1H), 4.54 (d, *J* = 4.5 Hz, 1H), 3.74 – 3.70 (m, 1H), 3.65 (t, *J* = 6.3 Hz, 1H), 3.63 – 3.59 (m, 1H), 3.56 (dt, *J* = 11.1, 5.5 Hz, 1H), 3.50 (dt, *J* = 11.4, 6.0 Hz, 1H), 3.47 – 3.41 (m, 1H), 3.20 (q, *J* = 6.7 Hz, 2H), 1.50 (t, *J* = 7.0 Hz, 2H), 1.34 – 1.19 (m, 14H), 0.86 (t, *J* = 6.9 Hz, 3H).

^{13}C-NMR (100 MHz, MeOD): δ 162.32, 161.12, 150.64, 132.29, 125.98, 116.70, 116.14, 102.59, 100.81, 75.82, 73.38, 70.68, 68.80, 61.03, 40.05, 31.65, 29.25, 29.02, 29.00, 28.97, 26.57, 22.31, 13.01.

HRMS (TOF MS ASAP+) (*m/z*): $[M+H]^+$ calcd. for $[C_{26}H_{37}N_2O_7+H]^+$ 491.2757, found 491.2737

FT IR (KBr) ν (cm^{-1}): 3522, 3296, 2920, 2850, 2216,1642, 1589 ,1533, 1514, 1263, 1187, 1089

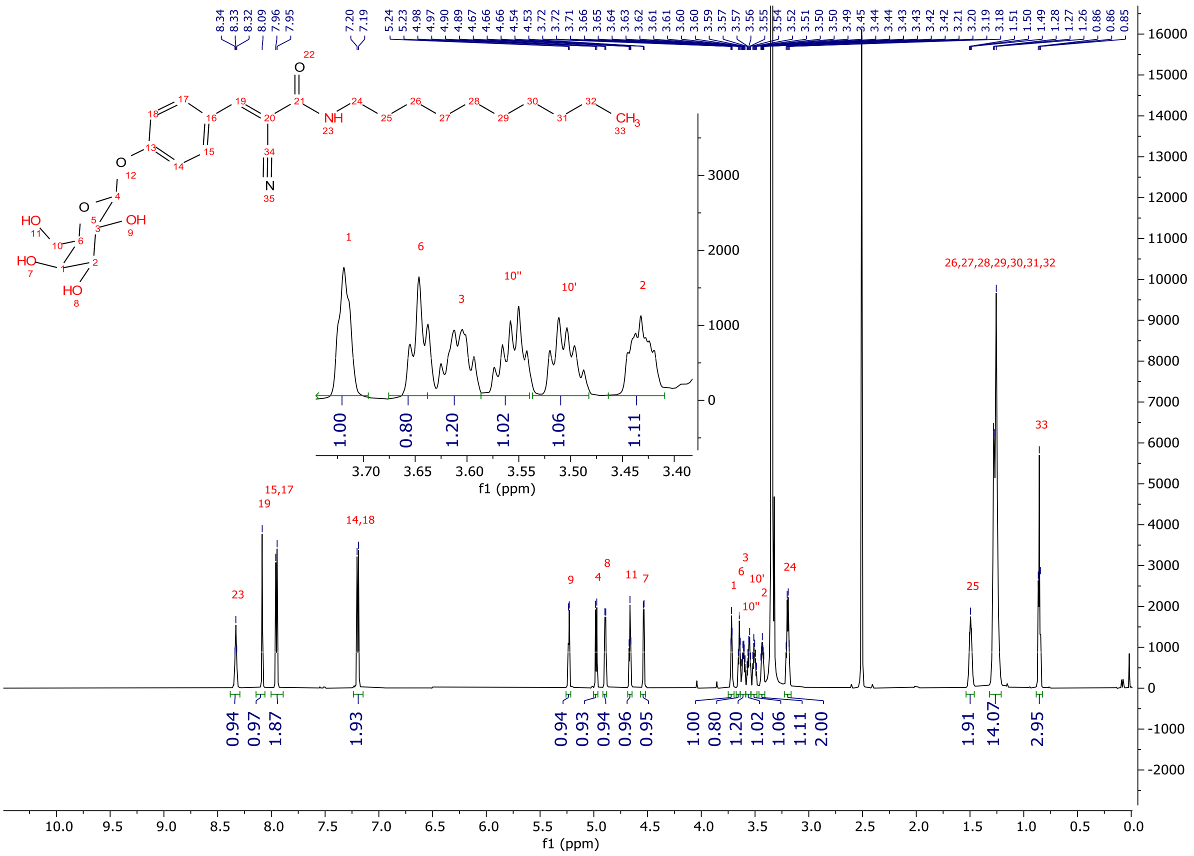

Figure S4 ^{1}H NMR (700 MHz, 301 K, DMSO-d_6) of compound **2**

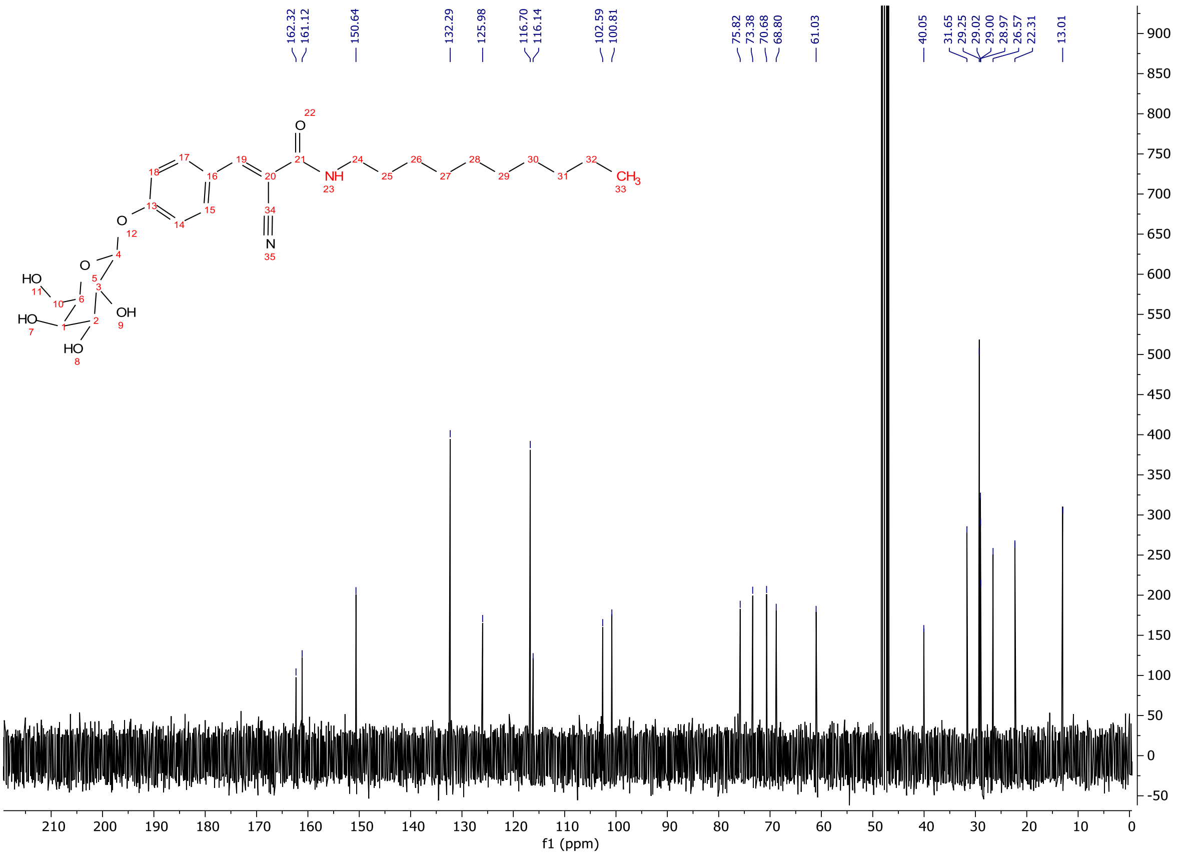

Figure S5 ^{13}C-NMR (100 MHz, 301 K, DMSO-d_6) of compound **2**

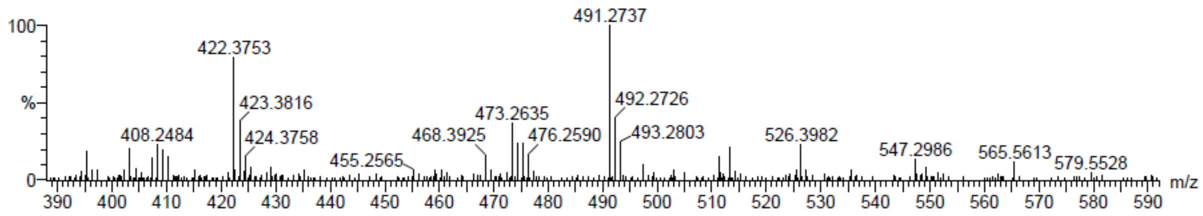

Figure S6 HRMS spectrum (ASAP+ TOF) (*m/z*) of **2**

3 General procedure for vesicles preparation

3.1 Loaded vesicles with 2 for UV-Vis titration and binding constant determination

A vesicle suspension was prepared in a LoB Eppendorf microcentrifuge tube by adding 1,2-dioleoyl-sn-glycero-3-phosphocholine (DOPC) in chloroform and receptor **2** from a methanol solution. The solvent was evaporated under nitrogen flow for 30 minutes until a dry lipid film was obtained. The lipid film was rehydrated with buffer (50 mM HEPES, 100 mM NaCl, pH=7.2) and sonicated for one minute. The solution was subjected to 4 freeze-thaw cycles using liquid nitrogen and a 40°C water bath. Subsequently the mixture was extruded 19 times through a 400 nm pore polycarbonate filter in an Avestin Lipofast apparatus. The extruded vesicles were eluted with buffer over a manual size-exclusion column (0.5 g Sephadex G-100, equilibrated with buffer. The vesicles were obtained with a final concentration of 0.50 mM with a 10% loading of **2** (0.05 mM). The loading of receptor **2** in the vesicles was confirmed by UV-Vis and HPLC.

3.2 Papain (and peptide) encapsulation for signalling experiments

A vesicle suspension was prepared in a LoB Eppendorf microcentrifuge tube by adding 1,2-dioleoyl-sn-glycero-3-phosphocholine (DOPC) in chloroform and receptor **1** or **2** from a methanol solution. The solvent was evaporated under nitrogen flow for 30 minutes until a dry lipid film was obtained. The lipid film was rehydrated with buffer (50 mM HEPES, 100 mM NaCl, pH=7.2), a 1 mg/mL solution of peptide (D-Ala-Leu-Lys-7-amido-4-methylcoumarin) in buffer and a 1 mg/mL solution of papain-$SSCH_3$ in buffer to yield a final concentration of 10 μM papain and 250 μM peptide. The solution was subjected to 4 freeze-thaw cycles using liquid nitrogen and a 40°C water bath. Subsequently the mixture was extruded 19 times through a 400 nm pore polycarbonate filter in an Avestin Lipofast apparatus. The extruded vesicles were eluted with buffer over a manual size-exclusion column (0.5 g Sephadex G-100, equilibrated with buffer). The vesicles were obtained with a final concentration of 1.0 mM with different loading of loading of receptor **1** [respectively in different preparation 0.1 mM (10%), 0.05 mM (5%) and 0.01 mM (1%)] and receptor **2** [0.01 mM (1%)], 10 μM papain and 250 μM peptide. One of the preparations was performed with no peptide encapsulated as a control experiment, the peptide was added externally. The loading of receptor **1** or **2** and the peptide in the vesicles was confirmed by UV-Vis and HPLC (Figure S13).

4 Kinetic Experiments and Binding Constant Determination for acceptor 2

4.1 UV-Vis spectra of thiol addition in Vesicles and Methanol

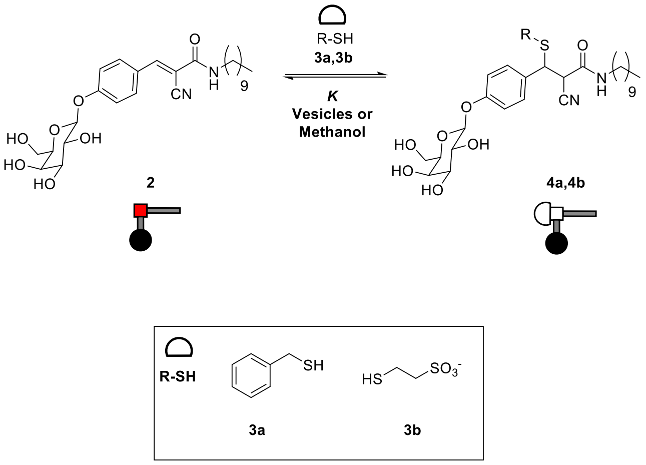

Scheme S1 Addition of thiols **3a,3b** to **2** leads to formation of the corresponding Michael adducts **4a,4b**.

Kinetics of thiols **3a,3b** addition to acceptor **2** embedded in vesicles were monitored with UV-Vis spectroscopy.

Vesicles

To vesicles formed by DOPC (0.5 mM, HEPES buffer, pH=7.2) loaded with receptor **2** (10% loading, 0.05 mM) were added different equivalent of thiols **3a,3b** in buffer from stock solutions at 5 mM concentration. UV-Vis spectra of kinetic experiments related to the addition of several equivalents of each thiol **3a,3b** to the system is reported in Figure S7.

Methanol

To **2** (0.075 mM) were added different equivalent of thiols **3a,3b** from 20 mM methanol solution. UV-Vis spectra of kinetic experiments related to the addition of several equivalent of each thiol **3a,3b** to the system is reported in Figure S8.

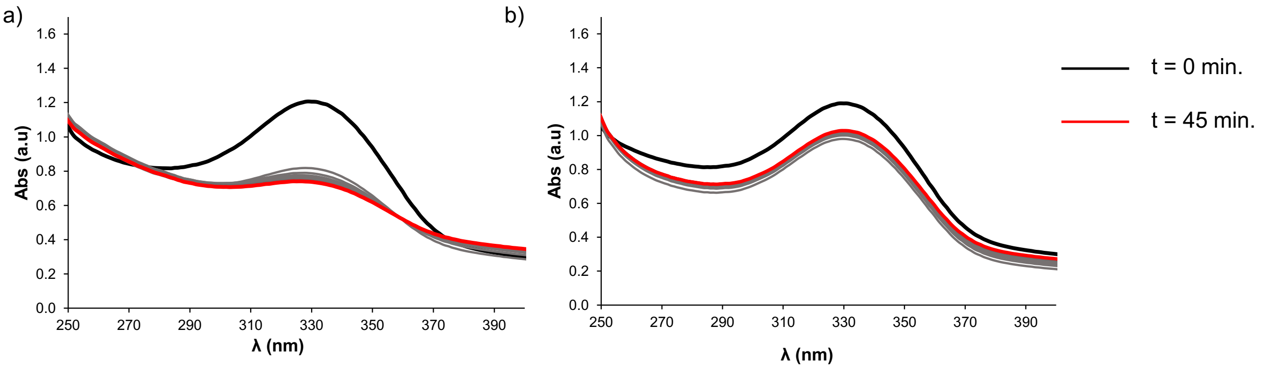

Figure S7 Selected UV-Vis spectra of adduct **4a,4b** formation. To the vesicles (0.5 mM, buffer) loaded with **2** (0.05 mM, 10% loading) black line) were added equivalents of thiols **3a** (2 eq.), **3b** (100 eq.).

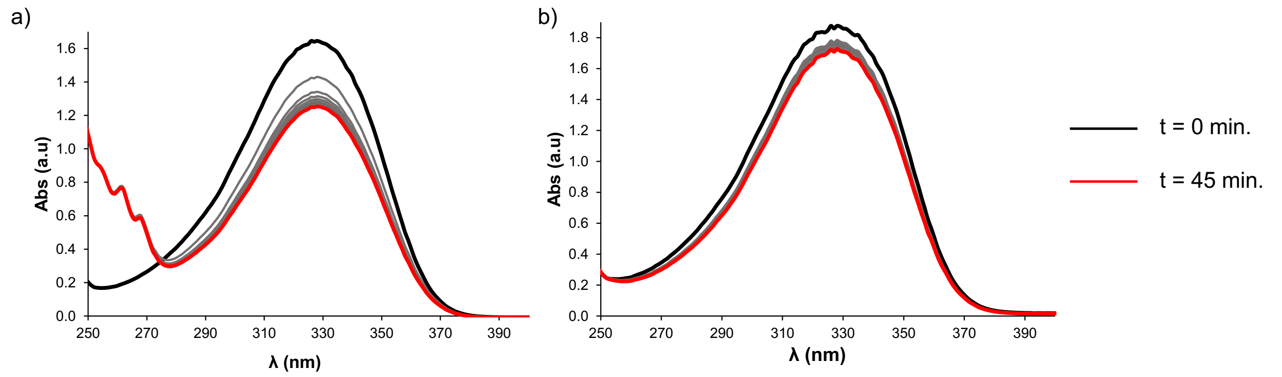

Figure S8 Selected UV-Vis spectra of adduct **4a** (a), **4b** (b) formation. To a methanol solution of **2** (0.075 mM, black line) were added 25 eq. (1.88 mM) of different thiols **3a,3b**.

4.2 Kinetic profile of the 2 adduct formation and binding constant determination

The formation of the adducts was monitored taking into account that the absorbance of **1** at 300 nm is quenched after addition of thiols,[1] adopting the same procedure as reported in our previous work.[2]

In Table S1-S2 are reported the concentration values for each species at the equilibrium when the adducts were formed in vesicles. In Table S3-S4 are reported the concentration values when the adducts were formed in methanol. In the last column of each table are reported the binding constant calculated at each equilibration point. The average binding constant value for each system is given with the related error and they are reported in Table S5 for both systems.

The kinetic trend for the formation of adducts **3a,3b** at different equivalents of thiols added with respect to **2** are reported using vesicles (Figure S10-S14) and in methanol solution (Figure S15-S19).

Vesicles

$[\mathbf{3a}]_0/[\mathbf{2}]_0$	$[\mathbf{3a}]_0$ (mM)	$[\mathbf{2}]_0$ (mM)	[**4a**] (mM)	[**3**] (mM)	[**3a**] (mM)	**K** ($1*10^4$ M^{-1})
2	0.098	0.050	0.032	0.017	0.066	2.74
2	0.098	0.050	0.033	0.015	0.064	3.43
5	0.238	0.050	0.040	0.007	0.198	2.79
5	0.238	0.050	0.040	0.007	0.197	3.03

Table S1 Concentrations of all species at equilibrium as determined by UV-Vis reported for repeated thiol **3a** additions to DOPC vesicles (0.5 mM, buffer) with 10% **2** imbedded (0.05 mM, buffer).

$[\mathbf{3b}]_0/[\mathbf{2}]_0$	$[\mathbf{3b}]_0$ (mM)	$[\mathbf{2}]_0$ (mM)	[**4b**] (mM)	[**3**] (mM)	[**3b**] (mM)	**K** (M^{-1})
100	4.54	0.050	0.009	0.036	4.54	58
100	4.76	0.050	0.009	0.039	4.75	48
200	9.09	0.050	0.013	0.032	9.08	43
200	9.09	0.050	0.013	0.032	9.06	44

Table S2 Concentrations of all species at equilibrium as determined by UV-Vis reported for repeated thiol **3b** additions to DOPC vesicles (0.5 mM, buffer) with 10% 2 imbedded (0.05 mM, buffer).

Methanol

[**3a**]$_0$/[**2**]$_0$	[**3a**]$_0$ (mM)	[**2**]$_0$ (mM)	[**4a**] (mM)	[**3**] (mM)	[**3a**] (mM)	**K** (M^{-1})
15	1.065	0.071	11.273	0.060	1.0538	179
15	1.065	0.071	11.175	0.060	1.0539	177
25	1.714	0.069	19.074	0.049	1.695	227
25	1.714	0.069	16.340	0.052	1.697	184

Table S3 Concentrations of all species at equilibrium as determined by UV-Vis reported for repeat thiol **3a** addition to **2** in methanol.

[**3b**]$_0$/[**2**]$_0$	[**3b**]$_0$ (mM)	[**2**]$_0$ (mM)	[**4b**] (mM)	[**3**] (mM)	[**3b**] (mM)	**K** (M^{-1})
15	1.065	0.071	2.995	0.068	1.062	41
15	1.065	0.071	1.928	0.069	1.063	26
25	1.714	0.069	3.845	0.065	1.710	34
25	1.714	0.069	5.211	0.063	1.709	48

Table S4 Concentrations of all species at equilibrium as determined by UV-Vis reported for repeated thiol **3b** additions to **2** in methanol.

**Adduct**	***K* MeOH (M^{-1}) - 2**	***K* Vesicles (M^{-1}) - 2**	***K* MeOH (M^{-1}) - 1**	***K* Vesicles (M^{-1}) - 1**
**4a**	169 ± 11	(3.00 ± 0.32)*10^4	800 ± 70	(2.07 ± 0.17)*10^5
**4b**	50 ± 3	49 ± 7	337 ± 6	236 ± 58

Table S5 Binding constants of thiols **3a,3b** with Michael acceptors **1** and **2** in MeOH and in the presence of vesicles. The values for receptor **1** were measured previously.[2]

4.2.1 Michael acceptor 2 kinetic traces in vesicles

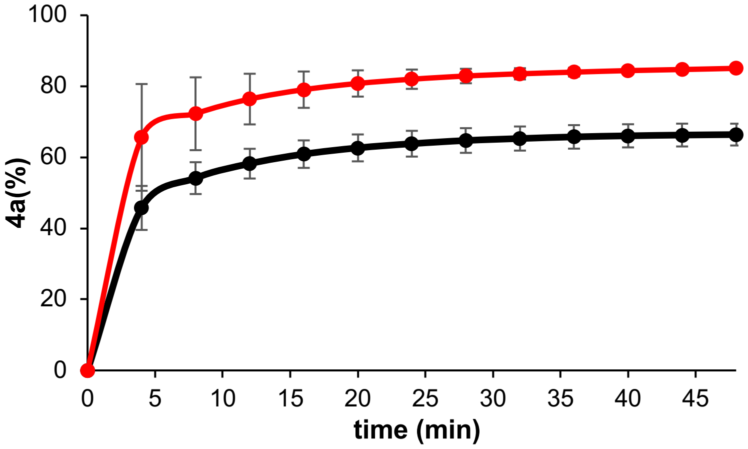

Figure S9 Kinetic trend for the formation of adducts **4a** within DOPC vesicles (0.5 mM) after the addition of thiol **3a**: — 2.0 eq.; — 4.0 eq. [**2**] =0.05 mM

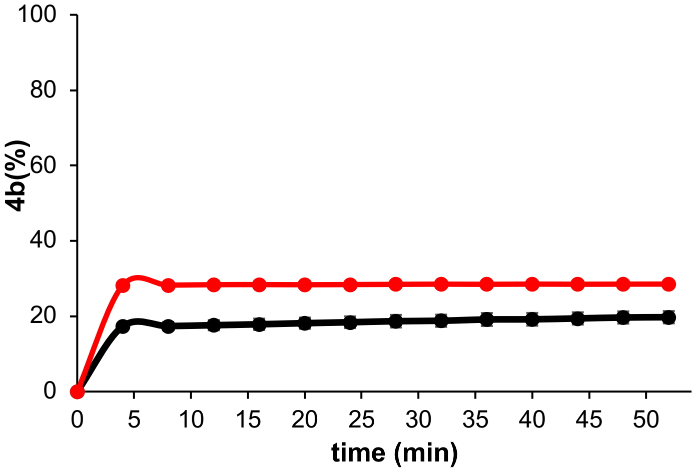

Figure S10 Kinetic trend for the formation of adducts **4b** within DOPC vesicles (0.5 mM) after the addition of thiol **3b:** — 200 eq.; — 100 eq. [**2**] =0.05 mM

4.2.2 Michael acceptor 2 kinetic traces in Methanol

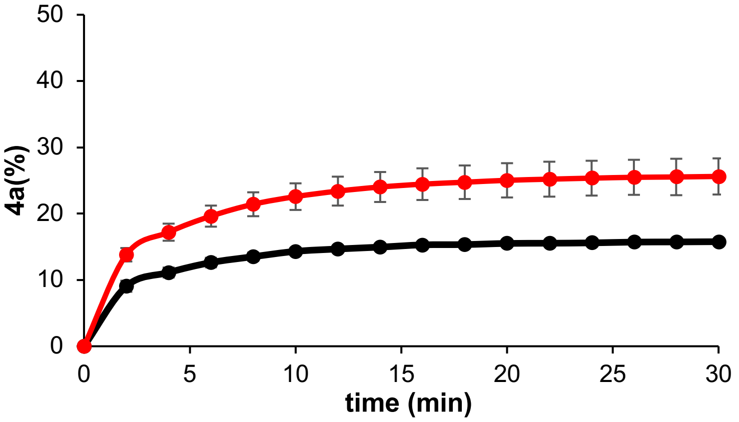

Figure S11 Kinetic trend for the formation of adducts **4a** in methanol after the addition of thiol **3a**: — 25.0 eq.; — 12.0 eq. [**2**] =0.075 mM

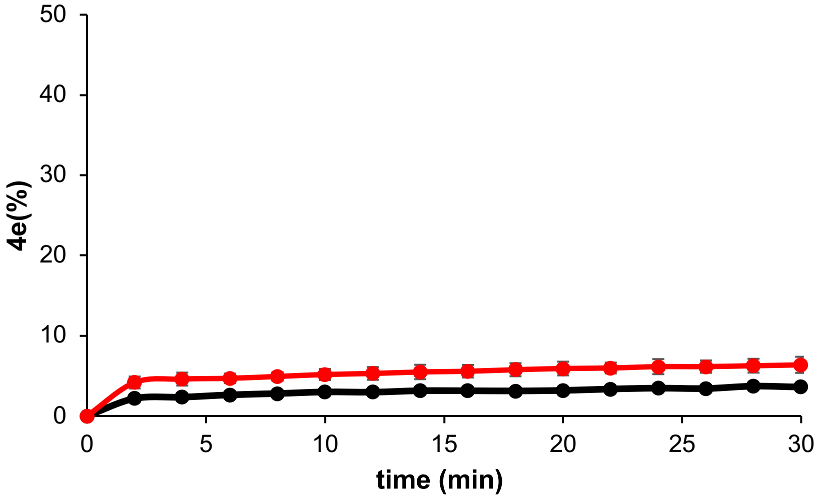

Figure S12 Kinetic trend for the formation of adducts **4b** in methanol after the addition of thiol **3b**: — 25.0 eq.; — 12.0 eq. [2] =0.075 mM

5 Loaded Vesicles Preparation

5.1 Purification of enzyme and peptide encapsulated vesicles

The HPLC traces of the fraction obtained after the GPC column confirms the presence of the Michael acceptors **1** or **2** together with the peptide substrate in the vesicles fraction respect to vesicles containing only peptide and enzyme. The other fractions collected show that the non-embedded compounds are retained in the column and the peptide is eluted after several fractions (Figure S13).

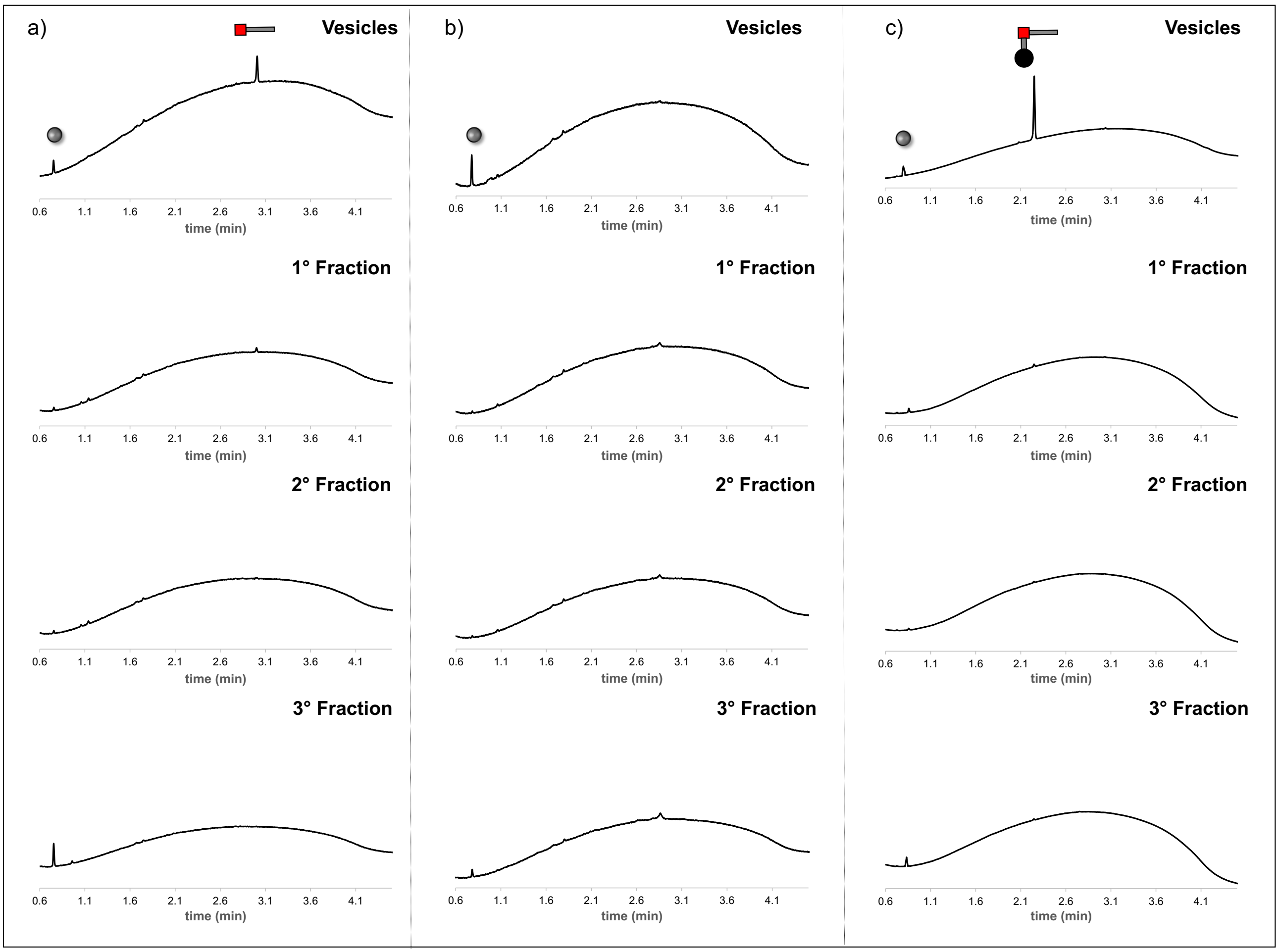

Figure S13 HPLC traces of different vesicles preparation. a) substrate, enzyme and **1**; b) substrate and enzyme; c) substrate, enzyme and **2.**

6 Signalling experiment controls

Signalling experiment were performed using a microplate reader using the parameter as described in the general method section. The data were normalized for the maximum fluorescence intensity of the reader.

6.1 Assessment of vesicles leakage upon thiols addition

Addition of thiols to vesicles loaded only with enzyme does not produce a significant change in fluorescence intensity if the substrate is present externally (Figure S14 for receptor **1**, Figure S15 for receptor **2**), confirming the system stability upon thiol addition. After the addition of a detergent the vesicles leak, therefore the active enzyme can interact with the substrate in solution.

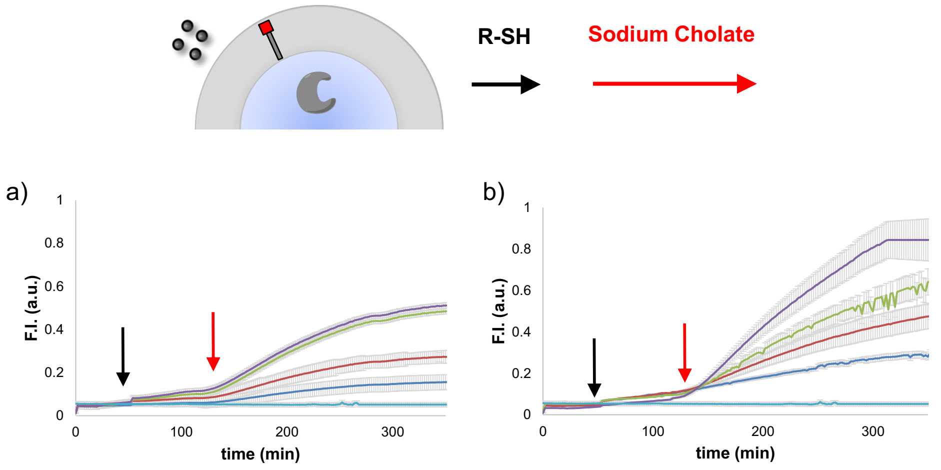

Figure S14. Time dependence of the relative fluorescence emission intensity at 440 nm (λ_{exc} = 365 nm). Vesicles (DOPC, 1.0 mM) are prepared in HEPES buffer (pH=7.2) papain (10 µM), **1** (0.1 mM, 10% loading). Substrate was added externally after vesicles preparation (10 µM). Thiols were added after 20 min (black arrow). Detergent sodium cholate hydrate (10%) was added after 120 min (red arrow). a) benzylmercaptane **3a**, b) mercaptoethansulphonate **3b.** [**Thiol**] = — 1000 µM, — 500 µM, — 100 µM, — 20 µM, — blank.

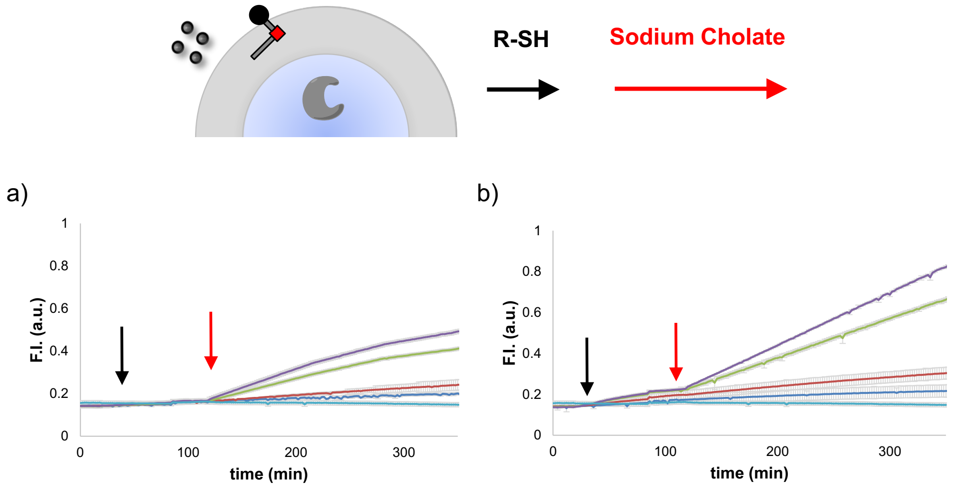

Figure S15. Time dependence of the relative fluorescence emission intensity at 440 nm (λ_{exc} = 365 nm). Vesicles (DOPC, 1.0 mM) are prepared in HEPES buffer (pH=7.2) papain (10 µM), **2** (0.01 mM, 1% loading). Substrate was added externally after vesicles preparation (10 µM). Thiols were added after 20 min (black arrow). Detergent sodium cholate hydrate (10%) was added after 120 min (red arrow). a) benzylmercaptane **3a**, b) mercaptoethansulphonate **3b.** [**Thiol**] = — 1000 µM, — 500 µM, — 100 µM, — 20 µM, — blank.

6.2 Reactivity between different thiols and enzyme

The thiols were reacted with the protected S-SMe papain. Addition of thiol at different concentration shows that the enzyme displays the same reactivity for the thiols (Figure S17). In Figure S16 is reported the variation of fluorescence intensity after 350 min at different thiol concentration.

In addition, benzyl thiol induce a deactivation of the enzyme respect to the other thiols (Figure S16a). This is showed by the fluorescence intensity plateau that is reached around after 100 min. after the thiol addition. This evidence is supported also in section S6.3.

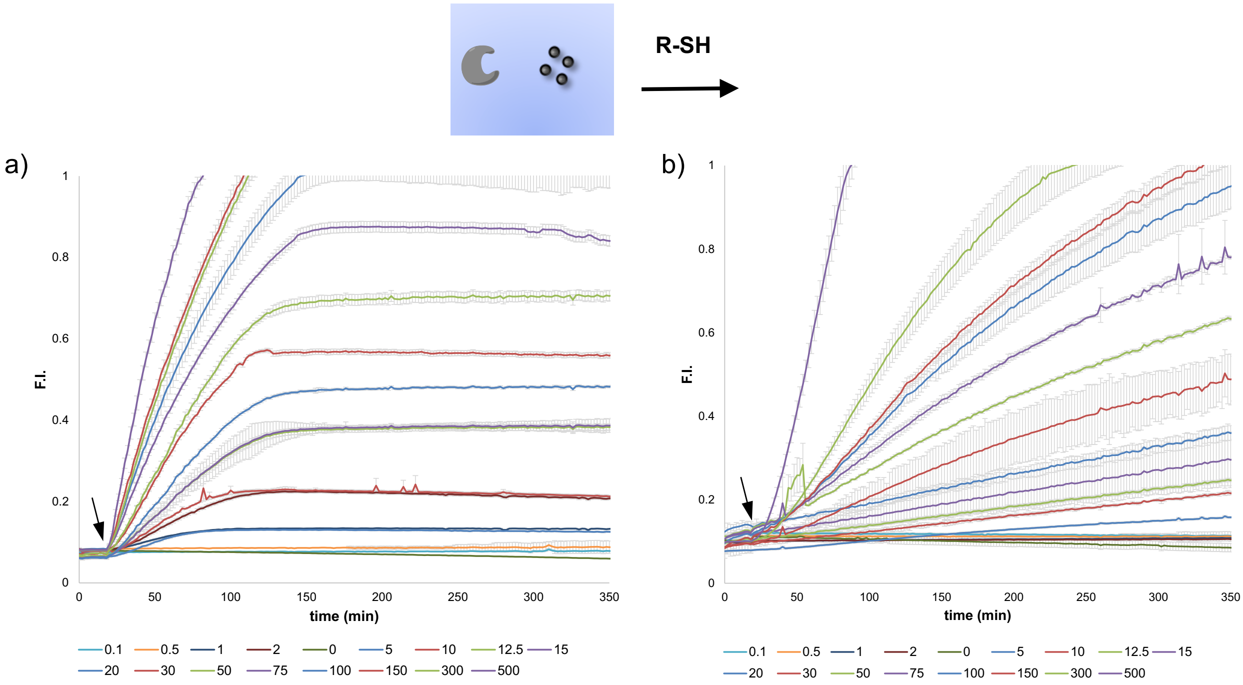

Figure S16. Time dependence of the relative fluorescence emission intensity at 440 nm (λ_{exc} = 365 nm). The system is composed by HEPES buffer (pH=7.2) papain (0.02 μM) and substrate (10 μM). Thiols were added after 20 min. a) benzylmercaptane **3a**, b) mercaptoethansulphonate **3b**. The thiol concentration is reported in the legend (μM).

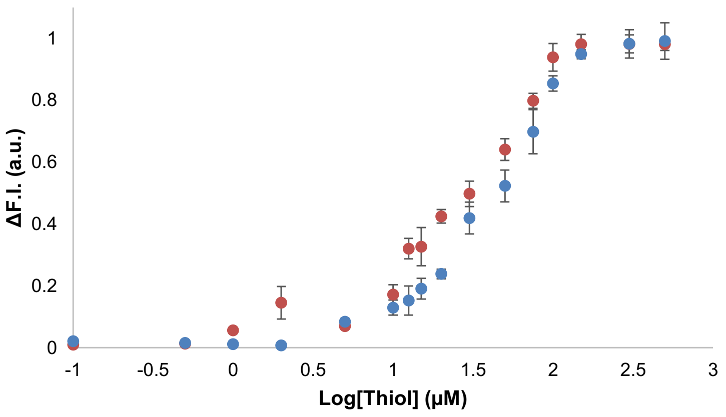

Figure S17. Normalized variation of fluorescent intensity (ΔF.I.) against thiol concentration.[●] = benzylmercaptane **3a**, [●] = mecaptoethansulphonate **3b**

6.3 System response with thiols

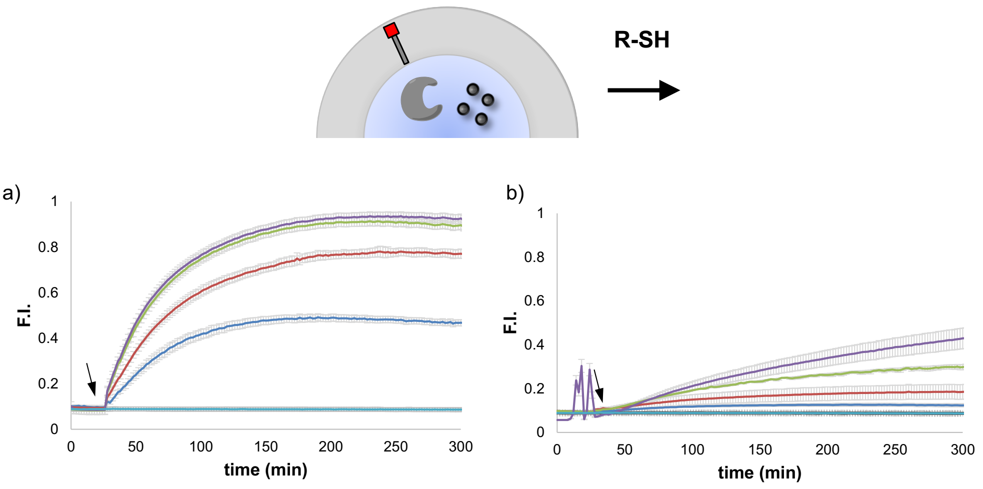

Figure S18. Time dependence of the relative fluorescence emission intensity at 440 nm (λ_{exc} = 365 nm). Vesicles (DOPC, 1.0 mM) are prepared in HEPES buffer (pH=7.2) papain (10 µM), **1** (0.1 mM, 10% loading), substrate (250 µM). Thiols were added after 20 min (black arrow). a) benzylmercaptane **3a**, b) mercaptoethansulphonate **3b**. [**Thiol**] = — 1000 µM, — 500 µM, — 100 µM, — 20 µM, — blank.

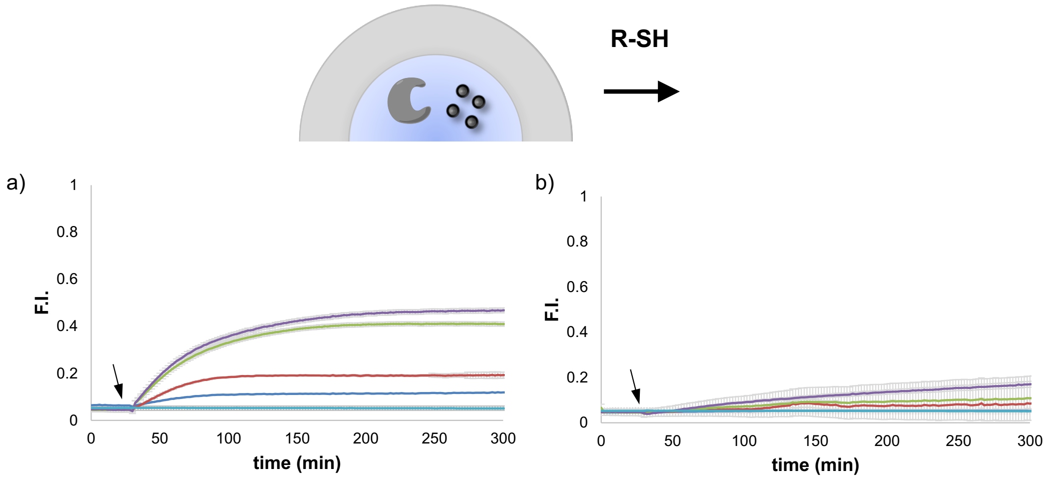

Figure S19. Time dependence of the relative fluorescence emission intensity at 440 nm (λ_{exc} = 365 nm). Vesicles (DOPC, 1.0 mM) are prepared in HEPES buffer (pH=7.2) papain (10 µM), substrate (250 µM). Thiols were added after 20 min (black arrow). a) benzylmercaptane **3a**, b) mercaptoethansulphonate **3b**. [**Thiol**] = — 1000 µM, — 500 µM, — 100 µM, — 20 µM, —blank.

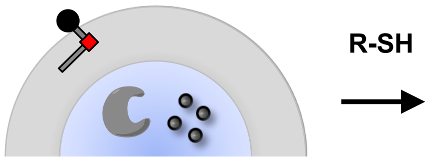

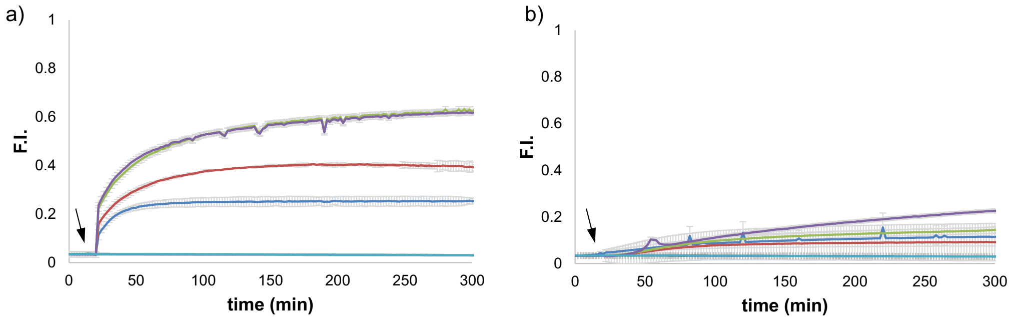

Figure S20. Time dependence of the relative fluorescence emission intensity at 440 nm (λ_{exc} = 365 nm). Vesicles (DOPC, 1.0 mM) are prepared in HEPES buffer (pH=7.2) papain (10 µM), **1** (0.01 mM, 1% loading), substrate (250 µM). Thiols were added after 20 min (black arrow). a) benzylmercaptane **3a**, b) mercaptoethansulphonate **3b**. [**Thiol**] = — 1000 µM, — 500 µM, — 100 µM, — 20 µM, — blank.

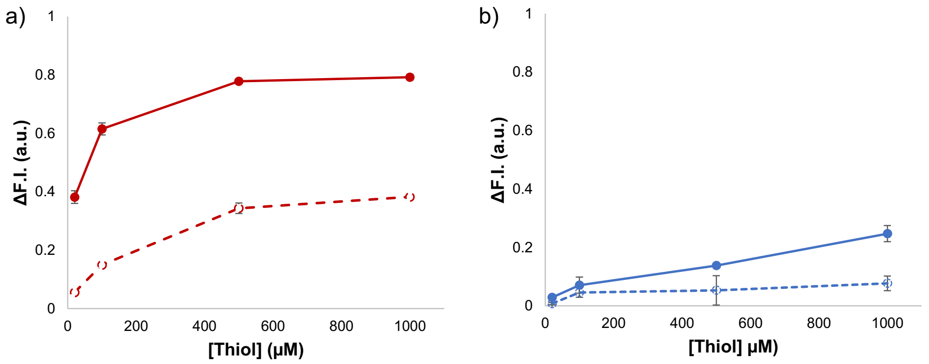

Figure S21. Normalized variation of fluorescent intensity (ΔF.I.) against thiol concentration. Vesicles (DOPC, 1.0 mM) are prepared in HEPES buffer (pH=7.2) papain (10 µM), substrate (250 µM). Vesicles loaded with 10% of acceptor **1** (0.10 mM): [●] = benzylmercaptane **3a**, [●] =mercaptoethansulphonate **3b.** Vesicles without receptor: [○] = benzylmercaptane **3a**, [○] = mercaptoethansulphonate **3b.**

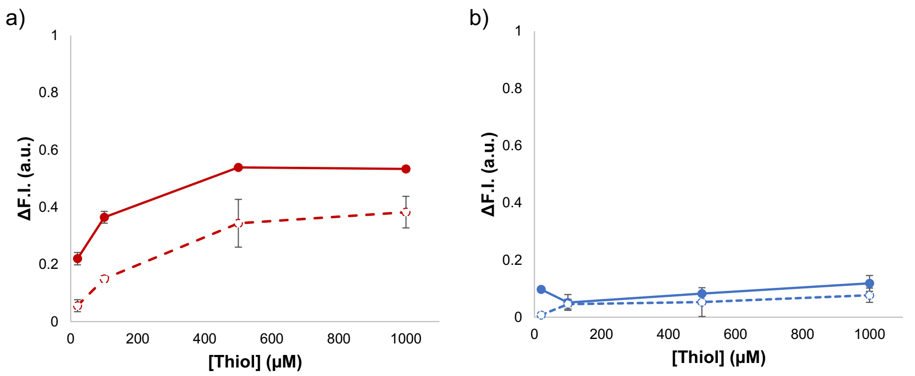

Figure S22. Normalized variation of fluorescent intensity (ΔF.I.) against thiol concentration. Vesicles (DOPC, 1.0 mM) are prepared in HEPES buffer (pH=7.2) papain (10 µM), substrate (250 µM). Vesicles loaded with 1% of acceptor **2** (0.01 mM): [●] = benzylmercaptane **3a**, [●] =mercaptoethansulphonate **3b.** Vesicles without receptor: [○] = benzylmercaptane **3a**, [○] = mercaptoethansulphonate **3b.**

6.4 Signalling experiment at different loading of Michael acceptors and binding constant evaluation of thiol 3a

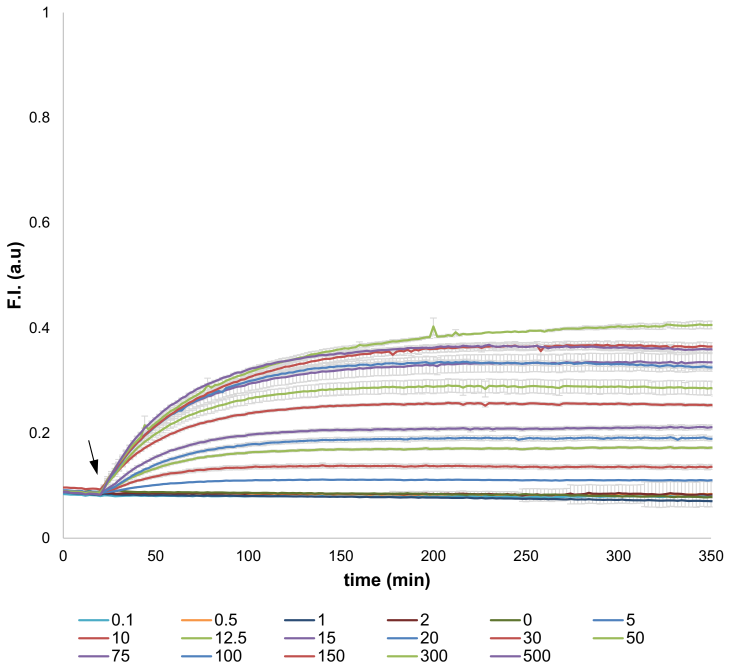

Figure S23. Time dependence of the relative fluorescence emission intensity at 440 nm (λ_{exc} = 365 nm). Vesicles (DOPC, 1.0 mM) are prepared in HEPES buffer (pH=7.2) papain (10 µM), substrate (250 µM), **1** (0.01 mM, 1% loading). Thiol was added after 20 min. The thiol concentration is reported in the legend (µM).

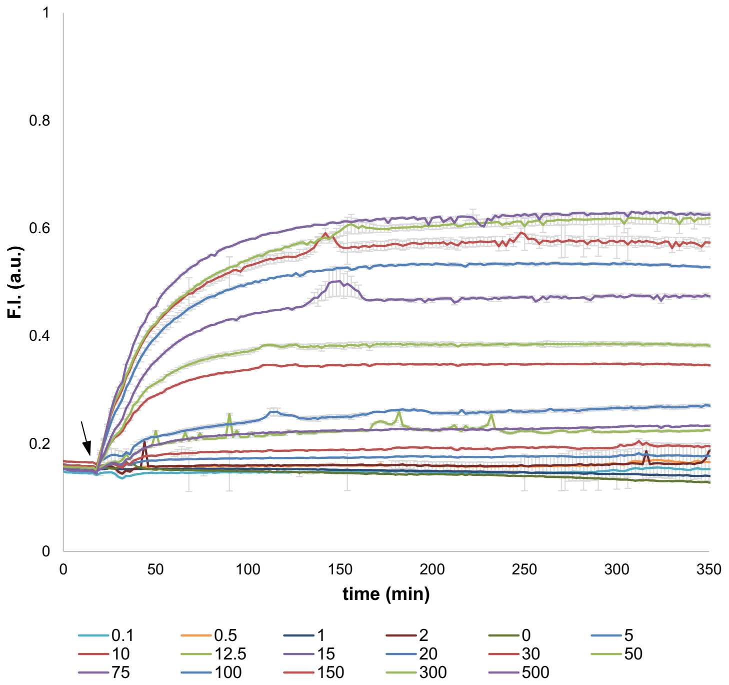

Figure S24. Time dependence of the relative fluorescence emission intensity at 440 nm (λ_{exc} = 365 nm). Vesicles (DOPC, 1.0 mM) are prepared in HEPES buffer (pH=7.2) papain (10 µM), substrate (250 µM), **1** (0.05 mM, 5% loading). Thiol was added after 20 min. The thiol concentration is reported in the legend (µM).

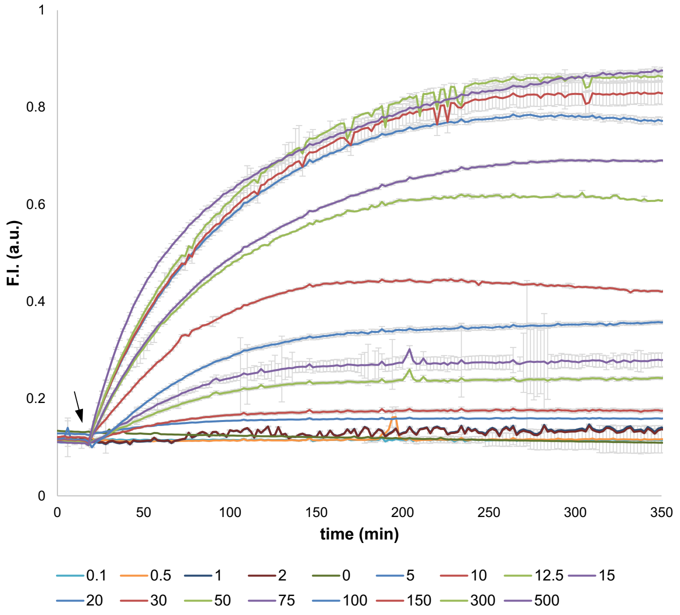

Figure S25. Time dependence of the relative fluorescence emission intensity at 440 nm (λ_{exc} = 365 nm). Vesicles (DOPC, 1.0 mM) are prepared in HEPES buffer (pH=7.2) papain (10 µM), substrate (250 µM), **1** (0.1 mM, 10% loading). Thiol was added after 20 min. The thiol concentration is reported in the legend (µM).

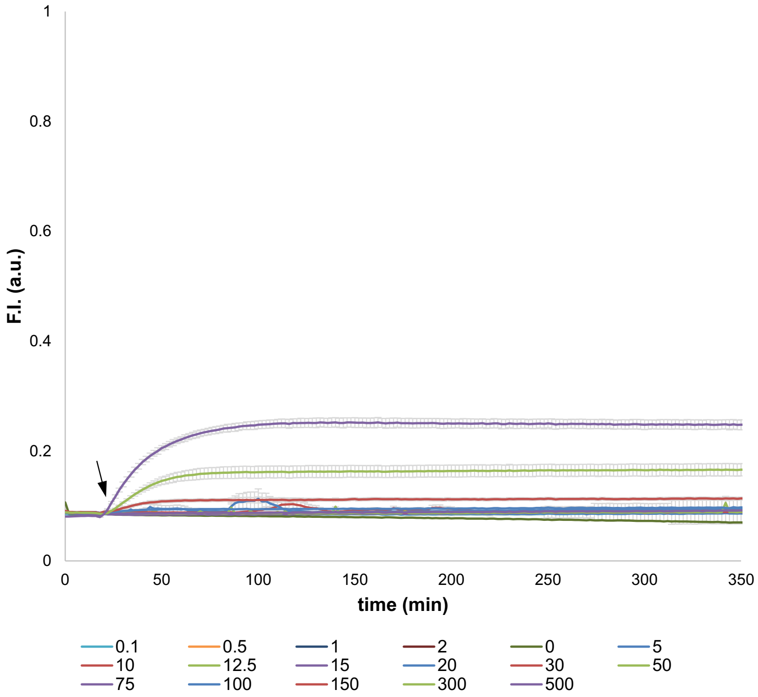

Figure S26. Time dependence of the relative fluorescence emission intensity at 440 nm (λ_{exc} = 365 nm). Vesicles (DOPC, 1.0 mM) are prepared in HEPES buffer (pH=7.2) papain (10 µM), substrate (250 µM). Thiol was added after 20 min. The thiol concentration is reported in the legend (µM).

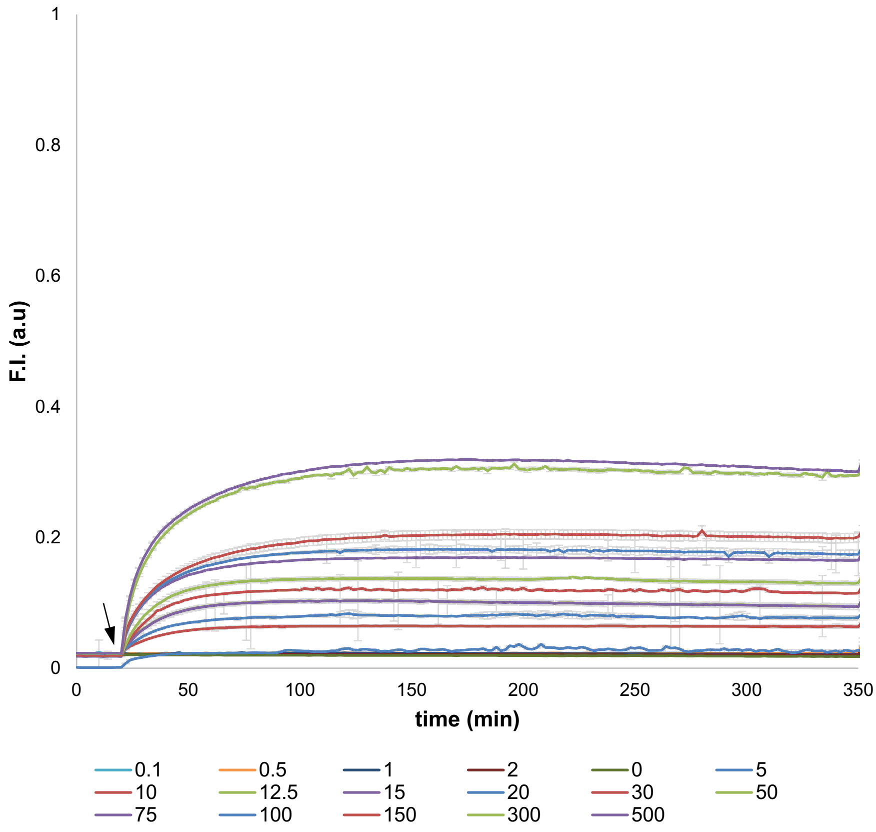

Figure S27. Time dependence of the relative fluorescence emission intensity at 440 nm (λ_{exc} = 365 nm). Vesicles (DOPC, 1.0 mM) are prepared in HEPES buffer (pH=7.2) papain (10 µM), substrate (250 µM), **2** (0.01 mM, 1% loading). Thiol was added after 20 min. The thiol concentration is reported in the legend (µM).

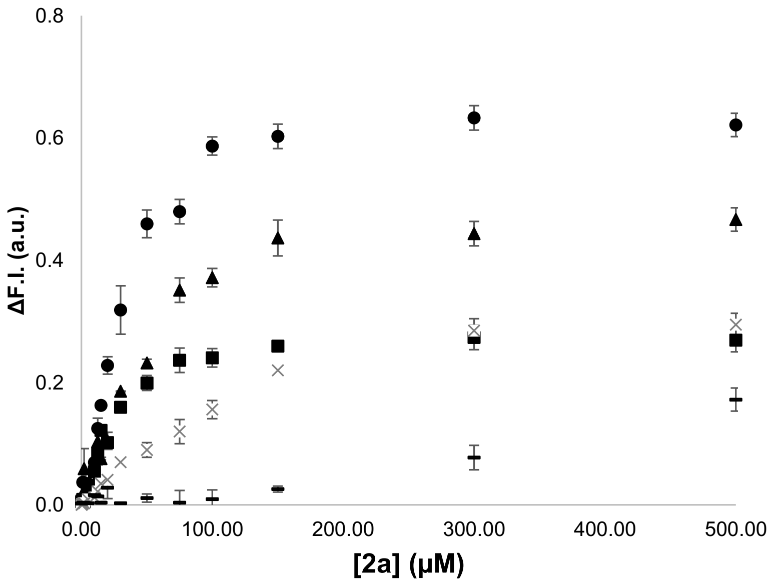

Figure S28. Experimental values of normalized variation of fluorescent intensity (ΔF.I.) between 20 and 360 min. against thiol concentration at different loading of Michael acceptor **1** and **2** in vesicles. [**1**] = 100 µM (10% loading) [●]; 50 µM (5% loading) [▲]; 10 µM (1% loading) [■], [**2**]: =10 µM (1% loading) [X], unloaded vesicles [—].Vesicles (DOPC, 1.0 mM) are prepared in HEPES buffer (pH=7.2), substrate (250 µM), papain (10 µM).

Fitting of the experimental data is reported as Figure 4 in the manuscript.

6.5 Papain deactivation

In order to establish the deactivation of papain due to benzyl thiol, a series of experiment have been devised. These experiments address the origin of the observed plateau for the fluorescence intensity when the enzyme is reacted with benzyl thiol.

The side reactivity of the generated coumarin derivative was proved by reacting 7-Amino-4-methylcoumarin with the thiol series at different concentration and monitoring the fluorescence emission. No significant changes in the fluorescent intensity were detected, therefore the plateau observed cannot be addressed to side reactivity of the fluorescent product with the thiols, especially benzyl thiol.

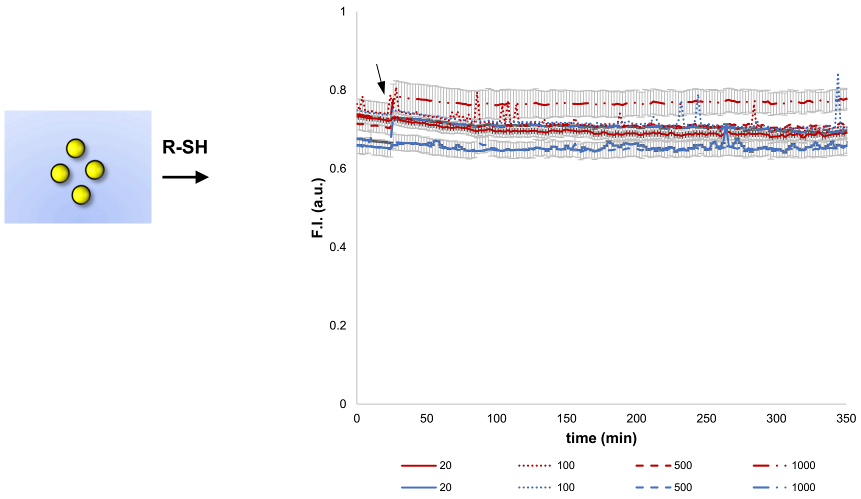

Figure S29. Time dependence of the relative fluorescence emission intensity at 440 nm (λ_{exc} = 365 nm) of amino coumarine derivative. To coumarine derivative (10 μM) in HEPES buffer (pH=7.2) were added thiol **3a** [●] and **3b** [●] after 20 min (black arrow). The thiols concentration is reported in the legend (μM).

The plateau observed in the case of benzyl thiol during signalling experiment has not been attributed to thiol oxidation. As shown in Figure S30 by adding the enzyme at different time interval to a solution containing the thiol, the fluorescence intensity observed is similar, hence concluding that minor eventual oxidation of the thiol is not the cause for the enzyme to stop working.

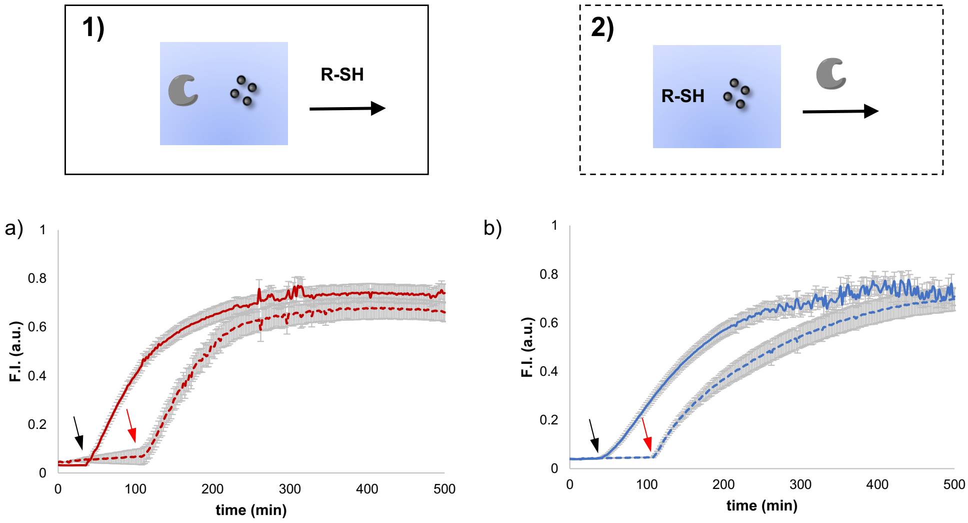

Figure S30. Time dependence of the relative fluorescence emission intensity at 440 nm (λ_{exc} = 365 nm). 1) First setup, to a solution of substrate (10 μM) and enzyme (0.02 μM) in HEPES buffer (pH=7.2) were added thiol **3a** [—], **3b** [—] after 20 min (black arrow) at 100 μM. 2) Second setup, to a solution of substrate (10 μM) and thiols (100 μM) were added the enzyme (0.02 μM) after 120 min (red arrow) in HEPES buffer (pH=7.2); thiol **3a** [---], **3b** [---].

In the experiment showed in Figure S31, the addition of **3a** in a competition experiment with thiol **3b** to papain display the previously observed plateau effect. In this case, after a first addition of thiol **3b** after 20 min. (black arrow) a second addition of thiol (red arrow): **3a** (red line) or **3b** (black line) led to different outcomes. For **3a**, after an initial burst the reaction stops after 100 min reaching a plateau, hence demonstrating that thiol **3a** reversibly inhibits the enzyme. As comparison for **3b**, the further addition of this thiol increase the enzymatic activity.

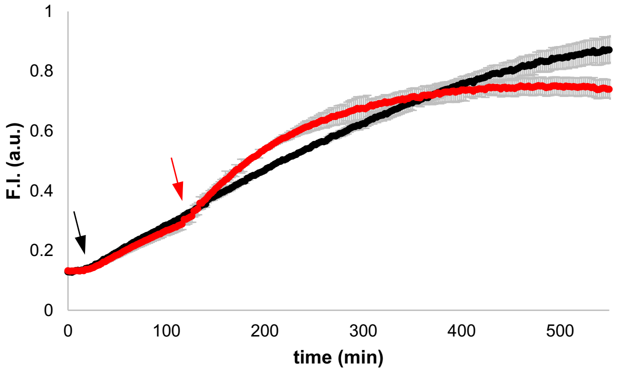

Figure S31. Time dependence of the relative fluorescence emission intensity at 440 nm (λ_{exc} = 365 nm). To a solution of substrate (10 μM) and enzyme (0.02 μM) in HEPES buffer (pH=7.2) were added thiol **3b** (10 μM) after 20 min. (black arrow). A second addition was made after 120 min. (red arrow) of thiol **3b**: [—] = 10 μM; **3a**: [—] = 10 μM.

6.6 Multi step papain activation

The cycles of transient activation for the enzyme in the presence of benzyl thiol (Figure S38) was demonstrated in the case of vesicles loaded with acceptor **1** (a), unloaded vesicles (b), and the system composed by enzyme and substrate (c). In Figure S32 is showed how the presence of **1** enhance the signalling in vesicles and how the transient activation is a specific property of the enzyme and benzyl thiol.

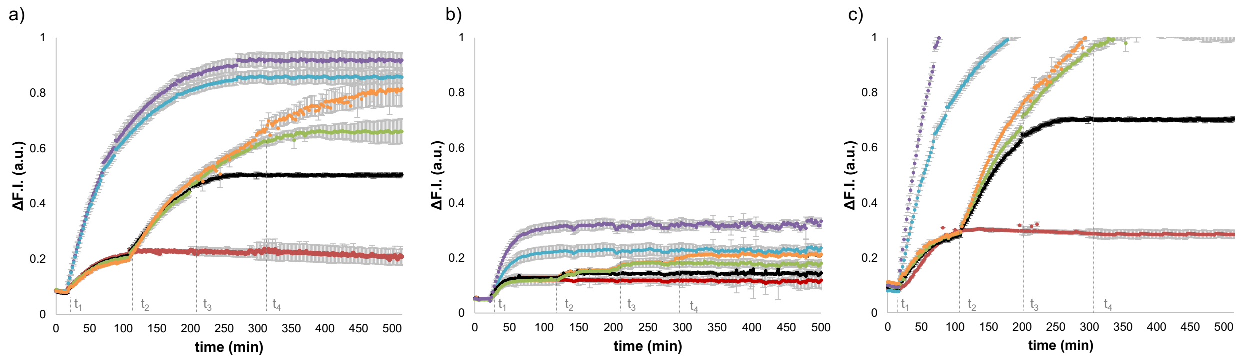

Figure S32. Transient system activation by multiple addition of **2a**. Time dependence of the relative fluorescence emission intensity at 440 nm (λ_{exc} = 365 nm). Benzylmercaptane **2a** was added in different aliquot at different time interval to different systems. a) aqueous solution of vesicles (DOPC, 1.0 mM) containing 0.1 mM **1** (10% loading) in HEPES buffer (pH=7.2), substrate (250 µM), papain (10 µM). (—, 25 µM-t_1); (—, 25 µM-t_1 + 25 µM-t_2); (—, 25 µM-t_1 + 25 µM-t_2 + 25 µM-t_3); (—, 25 µM-t_1 + 25 µM-t_2 + 25 µM-t_3 + 25 µM-t_4); (—, 100 µM-t_1); (—, 200 µM-t_1). b) aqueous solution of vesicles (DOPC, 1.0 mM) in HEPES buffer (pH=7.2), substrate (250 µM), papain (10 µM). (—, 25 µM-t_1); (—, 25 µM-t_1 + 25 µM-t_2); (—, 25 µM-t_1 + 25 µM-t_2 + 25 µM-t_3); (—, 25 µM-t_1 + 25 µM-t_2 + 25 µM-t_3 + 25 µM-t_4); (—, 100 µM-t_1); (—, 200 µM-t_1). c) HEPES buffer (pH=7.2), substrate (10 µM), papain (0.02 µM). (—, 12.5 µM-t_1); (—,12.5 µM-t_1 + 12.5 µM-t_2); (—, 12.5 µM-t_1 + 12.5 µM-t_2 + 12.5 µM-t_3); (—,12.5 µM-t_1 + 12.5 µM-t_2 + 12.5 µM-t_3 + 12.5 µM-t_4); (—, 50 µM-t_1); (—, 100 µM-t_1). t_1 = 20 min, t_2 = 120 min, t_3 = 220 min, t_4 = 320 min.

References

1 I. M. Serafimova, M. A. Pufall, S. Krishnan, K. Duda, M. S. Cohen, R. L. Maglathlin, J. M. McFarland, R. M. Miller, M. Frödin and J. Taunton, *Nat. Chem. Biol.*, 2012, **8**, 471–476.

2 C. Bravin and C. A. Hunter, *Chem. Sci.*, 2020, **11**, 9122–9125.
